# Quantitative proteomic analysis reveals hub proteins for high temperature-induced male sterility in bread wheat (*Triticum aestivum* L.)

**DOI:** 10.3389/fpls.2024.1426832

**Published:** 2024-09-03

**Authors:** Hongzhan Liu, Jinlei Li, Liuyong Xie, Huanhuan Wu, Shuying Han, Lizong Hu, Fuli Zhang, Hongxing Wang

**Affiliations:** ^1^ College of Life Science and Agronomy, Zhoukou Normal University, Zhoukou, Henan, China; ^2^ Field Observation and Research Station of Green Agriculture in Dancheng County, Zhoukou Normal University, Zhoukou, Henan, China; ^3^ Engineering Technology Research Center of Crop Molecular Breeding and Cultivation in Henan Province, Zhoukou Normal University, Zhoukou, Henan, China

**Keywords:** phenylpropanoid synthesis, reactive oxygen species, starch and sucrose metabolism, phosphatidylinositol signaling system, qRT-PCR

## Abstract

High-temperature (HT) stress can induce male sterility in wheat; however, the underlying mechanisms remain poorly understood. This study examined proteomic alterations across three developmental stages between normal and HT-induced male-sterile (HT-ms) anthers in wheat. Utilizing tandem mass tags-based proteomics, we identified 2532 differentially abundant proteins (DAPs): 27 in the tetrad stage, 157 in the binuclear stage, and 2348 in the trinuclear stage. Analyses through Gene Ontology and Kyoto Encyclopedia of Genes and Genomes pathways indicated significant enrichment of these DAPs in seven pathways, namely phenylpropanoid biosynthesis, flavonoid biosynthesis, sphingolipid metabolism, MAPK signaling pathway, starch and sucrose metabolism, response to heat, and response to reactive oxygen species (ROS). Our results indicated the downregulation of DAPs associated with phenylpropanoid biosynthesis and starch and sucrose metabolism, which aligns with anther indehiscence and the lack of starch in HT-ms anthers. By contrast, DAPs in the ROS pathway were upregulated, which aligns with excessive ROS accumulation in HT-ms anthers. Additionally, we conducted protein–protein interaction analysis for the DAPs of these pathways, identifying 15 hub DAPs. The abundance of these hub proteins was confirmed through qRT-PCR, assessing mRNA expression levels of the corresponding transcripts. Collectively, these results offer insights into the molecular mechanisms underlying HT-induced male sterility in wheat at the proteomic level, providing a valuable resource for further research in plant sexual reproduction.

## Introduction

Wheat originated in specific regions within the Fertile Crescent and has since spread globally (Salamini et al., 2002). Currently, bread wheat (*Triticum aestivum* L.) is the world’s most widely grown crop and among the first to be domesticated, with a trade value that surpasses that of all other crops combined (Lafiandra et al., 2014). Because of their mineral, vitamin, and fat contents, wheat grains have served as a staple food for humans for millennia (Hawkesford et al., 2013; Lafiandra et al., 2014). With the rising global population, the demand for wheat is increasing rapidly, leading to a prominent supply–demand imbalance. By 2050, the global population is projected to exceed 9 billion, necessitating an approximately 70% increase in wheat production to meet the demands of the future population (Foley et al., 2011; Tilman et al., 2002). However, over the past decade, the increase in the yield of wheat has been unsatisfactory and concerning, which is attributable to three main factors. First, the usage rate of hybrid varieties in wheat production is lower than that of other crops such as *Zea mays* (corn) and *Oryza sativa* (rice). Second, the substantial deterioration of natural environmental factors, such as precipitation and temperature, has led to a significant decrease in wheat production owing to severe environmental changes during critical growth stages. Third, wheat is an allohexaploid crop with a large genome, which hinders the use of hybrid varieties with various benefits. However, harnessing the advantage of wheat hybrid is considered an effective method to improve its yield. Thus, the mechanism of male sterility, which serves as the foundation for leveraging heterosis in wheat, has been a prominent field of wheat research.

In the flowering plants, the male reproductive development process is categorized into three main stages: male meiosis (tetrad stage), microspore development (tapetum degeneration stage), and anther dehiscence (trinuclear stage) (Ma, 2005). Anther development is particularly more sensitive to abiotic stresses, such as heat stress, than to the availability of growth nutrients (De Storme and Geelen, 2014). Since the early 20th century, global warming has increased the average global temperature by approximately 1.5°C (Seneviratne et al., 2018). Elevated external ambient temperatures during sexual reproduction in plants can cause abnormal pollen development, decreased viability, and impaired germination and fertilization. In cotton and maize, high temperatures during the tetrad stage substantially hinder pollen development (Begcy et al., 2019; Masoomi-Aladizgeh et al., 2021). In barley, elevated temperatures lead to the degeneration of the tapetum, enhanced accumulation of reactive oxygen species (ROS) during microsporogenesis, and changes in the morphology of the tapetal rough endoplasmic reticulum (Lohani et al., 2020). In rice, high-temperature (HT) stress (35°C–38°C) during meiosis suppresses the expression of *cCu/Zn-SOD1* and *OsCATB* genes in the anthers, reducing the activities of superoxide dismutase (SOD) and catalase (CAT). This phenomenon, in turn, leads to a significant increase in ROS levels in the anthers, causing oxidative damage and membrane structure damage to the cells and subsequently reducing pollen viability. Therefore, enhancing SOD or CAT activity to effectively suppress ROS can enhance tolerance to HT stress during meiosis (Zhao et al., 2018a, 2018). In addition, HT stress accelerates programmed cell death (PCD) of the tapetum cells, disrupting interactions between microspores and the tapetum and thus affecting the development and function of pollens (Hedhly et al., 2009). In *Arabidopsis*, AtMYB80 encodes a transcription factor that mediates the normal degradation of the tapetum by regulating the expression of the A1 aspartic protease UNDEAD. Under HT conditions, the expression of AtMYB80 and UNDEAD is suppressed and the degradation of the tapetum is accelerated, thereby affecting pollen development and anther dehiscence (Dündar et al., 2019). Similarly, in HT-sensitive cotton material, HTs affect PCD in the tapetum and microspore development through the GhMYB66/GhMYB4-GhCKI pathway, eventually leading to male sterility (Li et al., 2022). Our previous transcriptomic study in wheat indicated the involvement of differentially expressed genes in starch and sucrose metabolism, PI signaling, peroxidase activity, oxidative stress response, and heme binding in HT-sterile anthers than in normal anthers (Liu et al., 2021a). In addition, the findings of the TUNEL assay confirmed the excessive accumulation of ROS in sterile anthers (Liu et al., 2021a). In our previous study on the PIP5K gene family of HT-induced male sterility in wheat, we examined alterations in gene expression, enzyme activity, and hormone metabolism (Liu et al., 2021b). However, the proteomic aspects of HT-induced wheat anther abortion remain unexplored.

Proteomics has become a favorable tool for exploring the dynamic behavior of proteins and their complex regulatory networks. Because mRNA expression alone does not fully reflect protein expression, proteomic analysis has increasingly gained prominence, becoming an essential method for examining cellular functionality (Nobori et al., 2020; Wang et al., 2020). Quantitative proteomics is a potent high-throughput tool that can be used to identify key proteins involved in the male sterility pathway and elucidateassociated molecular mechanisms. In recent years, proteomic techniques, such as tandem mass tags (TMT), have been used to examine anther development and pollen reproduction in various plant species, including *Arabidopsis thaliana*, soybean (*Glycine max*), barley (*Hordeum vulgare* L.), rice (*Oryza sativa* L.), cotton (*Gossypium* spp.), and tomato (*Solanum lycopersicum* L.) (Kim et al., 2015; Lewandowska et al., 2022; Li et al., 2023; Mazzeo et al., 2018; Mu et al., 2017; Wang et al., 2023, 2019; Wu Y. et al., 2019).

Proteomic studies on tomato anthers have revealed that heat stress primarily affects energy and amino acid metabolism and nitrogen assimilation. In addition, proteins such as glutamine synthetase, S-adenosylmethionine synthetase, and polyphenol oxidase were identified as potentially associated with heat tolerance traits (Mazzeo et al., 2018). Moreover, proteomic analysis of the tomato anther tetrad stage in the 7B-1 male sterility mutant indicated a reduction in the levels of proteasome and 5B proteins associated with tapetum degradation, suggesting their involvement in male sterility (Sheoran et al., 2009). In the thermosensitive genetic male-sterile line AnnongS-1, 89 proteins with differential abundance were identified in sterile samples. Gene Ontology (GO) analyses of these proteins demonstrated that HTs hinder pollen growth and development by reducing the levels of crucial proteins involved in defense and resilience, particularly affecting protein, carbohydrate, and energy metabolism (Wang et al., 2019). In cotton, proteins differentially expressed in sterile anthers are mainly associated with pyruvate, carbohydrate, and fatty acid metabolism (Wu Y. et al., 2019). Similarly, in rapeseed (*Brassica napus* L.), proteins linked to carbohydrate and energy metabolism, photosynthesis, and flavonoid synthesis were downregulated in sterile anthers, indicating their involvement in anther sterility (Sheoran and Sawhney, 2010). In wheat, proteomic analysis of the thermosensitive sterile line YS3038-A revealed significant reductions in the soluble sugar and ATP levels, a notable increase in free fatty acid levels, and abnormal ROS accumulation (Ma et al., 2022). Overall, these proteomic studies have shown that heat stress significantly influences multiple biochemical pathways in anther development of different plant species, highlighting the complexity and importance of these processes in anther male sterility.

This study elucidated functional mechanisms underlying HT-induced male sterility. We used tandem mass tags (TMT) proteomics to examine differences in protein expression between HT-induced sterile anthers and normal anthers at various developmental stages. In addition, we conducted a comprehensive analysis of the effects of HT on male steriity by integrating the data on these differentially expressed proteins with observations from phenotypic traits, paraffin sectioning, starch iodine staining, and scanning electron microscopy of sterile anthers. Our study offers a new perspective to understand the occurrence of HT-induced male sterility in wheat, laying a basis for the development of male-sterile lines or HT-resistant lines through targeted breeding programs.

## Materials and methods

### Wheat material, phenotype observations, and histological analyses

Wheat (*T. aestivum L.*) seeds of cv Zhoumai 36, provided by Zhoukou Academy of Agricultural Sciences, were sown on October 21, 2021, in the experimental fields of Zhoukou Normal University, situated in Zhoukou, Henan Province, People’s Republic of China (33°64′N, 114°68′E). We planted two plots in the experimental field, each plot including 20 rows of wheat. For both plots, a piece of transparent plastic film supported by thin steel pipes and plastic joints was set up to cover the wheat plants when necessary. When the wheat reached the Feekes growth stage 8.5, the external appearance manifested as the flag leaf being half the length of the penultimate leaf. The plants in one plot were covered with a transparent plastic film to induce high temperature, whereas those in the other plot were left uncovered as a control. The detailed experimental setup and HT stress treatments followed the methodologies described in previous studies (Liu et al., 2022). Each anther sample had three biological replicates. The samples were treated with formalin-acetic acid-ethanol fixative, and vacuum pumping was used to ensure thorough penetration of the fixative into the anther tissues. The anthers were then preserved in 70% alcohol at 4°C for no more than one week to be used for subsequent sectioning. For microscopic analysis, the fixed anthers were sectioned using the conventional paraffin technique at a thickness of 12 μm and stained with Safranin O-Fast Green (Liu et al., 2021b). The examination of pollen grains under a microscope was performed post their staining with a 1% I_2_-KI solution. For the purpose of electron microscopy, anthers and pollen grains underwent freeze-drying, were coated with palladium, and were then visualized using a scanning electron microscope (SEM) set to an acceleration voltage of 25 kV (Liu et al., 2021b).

### Protein extraction and digestion

Analysis was conducted on Normal anthers and HT-ms anthers at three stages (tetrad stage, binuclear stage, and trinuclear stage); Each sample was set with three biological replicates, totaling 18 samples. Anther samples (0.15g), previously frozen at - 80°C were placed in a pre-cooled grinding tube and ground into a powder using liquid nitrogen. Using the Plant Total Protein Extraction Kit (Bangfei Bioscience), the lysis buffer was added step by step according to the operating instructions, and the final supernatant collected served as the protein solution. The supernatant procured in the end was then stored at -80°C, awaiting further experimentation. Subsequently, the total protein quantity was determined using a microplate reader (H4MFPTAD, BioTek), and the corresponding concentration was deduced from the standard curve. The subsequent procedures of trypsin (Promega) digestion were strictly conducted according to the steps and operations described in reference (Ma et al., 2022). A 100 μg of each protein samples was mixed with dithiothreitol (DTT; Sigma) at a final concentration of 10 mM, incubate at 37°C for 1 hour and returned to room temperature. To each sample, iodoacetamide (Sigma) was added at a final concentration of 40 mM and samples were kept in the dark at room temperature for 45 minutes. The sample was diluted using ammonium bicarbonate. The pH was measured to be 8, and trypsin was added at a ratio of 50:1 in relation to the protein. The mixture was then kept at 37°C throughout the night. On the next day, 50 μl of 0.1% Formic acid (FA; Sigma) was brought in to halt the reaction. A C18 desalting column was utilized for desalting the samples. The column was balanced with 0.1% FA, the sample was loaded onto the column, then the column was washed with 0.1% FA to remove impurities, and finally eluted with 70% acetonitrile (Sigma). The flow-through liquid was collected and freeze-dried.

### TMT labeling

In this study, the Thermo Fisher Scientific TMT 10plex Isobaric Label Reagent (Thermo Scientific, A52047 and A44522) was employed to label 100 µg of the 18 samples, which comprised three stages in the Normal and HT-ms anthers, with three replicates each. The corresponding tags were 126, 127 N and 127 C for normal anthers at the tetrad stage; 128N, 128C and129N for HT-ms anthers at the tetrad stage; 129C, 130N and130C for normal anthers at the binuclear stage;131N, 131C, and 132N for HT-ms anthers at the binuclear stage; 132C, 133N and 133C for normal anthers at the trinuclear stage; 134N, 134C and 135N for HT-ms anthers at the trinuclear stage. The TMT reagent was brought to room temperature. An appropriate amount of peptide segments from each sample group was taken and labeled strictly according to the manufacturer’s TMT kit (Thermo Scientific) protocol. Subsequently, the dried labeled samples were redissolved with mobile phase solution and fractionated using a C18 column under high pH conditions via HPLC (Ross et al., 2004; Ma et al., 2022). The combined fractions were centrifuged at 14,000 g for 10 min and the supernatant was aspirated for mass spectrometry analysis.

### Peptide identification by nano UPLC–MS/MS

The nano UPLC-MS/MS system, which integrates a Nanoflow HPLC system (Thermo Scientific) with a Q Exactive HF-X mass spectrometer (Thermo Scientific), received an appropriate amount of supernatant for analysis. Utilizing a data-dependent acquisition mode, the mass spectrometry detected and scrutinized parent ions of peptides and their secondary fragments in full MS scan. The details are as follows: The mobile phase A solution (comprising 100% water and 0.1% formic acid) and B solution (containing 80% acetonitrile and 0.1% formic acid) were prepared. The lyophilized powder was dissolved using 10 µL of solution A, followed by centrifugation at 14000 g for 20 min at 4°C. Then, 1 µg of the supernatant sample was injected for liquid chromatography-mass spectrometry (LC-MS) analysis. The Q Exactive HF-X mass spectrometer and Nanospray Flex™ (NSI) ion source were employed. The ion spray voltage was set to 2.4 kV, and the ion transfer tube temperature was 275°C. The mass spectrometer adopted a data-dependent acquisition mode. The full scan range of the mass spectrometer was m/z 407-1500. The resolution of the primary mass spectrometer was set at 60000 (at 200 m/z), the AGC was 3×10^6^, and the maximum injection time of the C-trap was 20 ms. The top 40 parent ions with the highest ion intensity in the full scan were selected and fragmented using the high-energy collision dissociation method for secondary mass spectrometry detection. The resolution of the secondary mass spectrometer was set at 45000 (at 200 m/z), the AGC was 5×10^4^, the maximum injection time was 86 ms, and the peptide fragmentation collision energy was set at 32%. The IWGSC Annotation v. 1.1 database was utilized for this experiment. The MS/MS data obtained were analyzed using Proteome Discoverer 1.4 software. The identification parameters were set according to standard protocols, including precursor ion mass tolerance (± 10 ppm), fragment ion mass tolerance (± 0.02 Da), and maximum missed cleavages (2), among others.

### Identification of differentially abundant proteins and data bioinformatics analyses

For protein differential analysis, the sample pairs to be compared were initially picked out. The ratio of the average quantitative values of each protein across all biological replicates within the compared sample pairs was considered as the Fold Change (FC). For the determination of the significance of the variance, a Student’s t-test was performed on the relative quantitative values of each protein in the two compared sample pairs, and the corresponding P value was calculated as an indicator of significance. The identification process utilized the UniProt database alongside the *Triticum aestivum* (IWGSC) protein database. Proteins identified with at least a 1.90-fold change and a P value ≤0.02 were considered DAPs, where an increase was denoted by FC ≥ 1.9 and P ≤ 0.02, and a decrease by FC ≤ 1/1.9 and P ≤0.02. GO analysis was conducted using InterProScan software (Jones et al., 2014), and the protein family and pathway analyses were performed with the Kyoto Encyclopedia of Genes and Genomes (KEGG) databases (http://www.genome.jp/kegg/) (Kanehisa et al., 2016). The enrichment of pathways in both GO and KEGG was determined using the identified DAPs. The visualization of the expression heatmap was accomplished using the TBtools software (Chen et al., 2020). Protein–protein interaction (PPI) analysis was performed according to established methods and visualized using Cytoscape v. 3.9.1 software (Liu et al., 2021b; Kohl et al., 2011).

### Total RNA isolation and qRT−PCR validation analysis

Employing the TRIzol method, total RNA was isolated from the anther samples that were flash-frozen in liquid nitrogen. Upon validation of the RNA quality, cDNA was produced through reverse transcription using the RevertAid First Strand cDNA Synthesis Kit (Thermo Scientific). Specific primers for qRT-PCR experiments were designed using Primer Premier 5.0 software (Premier Biosoft International, Palo Alto, CA, USA). Furthermore, details regarding primer sequences, primer lengths, and product lengths are provided in **Supplementary Table S1**. The CFX Connect Real-Time system (BioRad) was used, and the reaction was run in a 20-µL volume containing 2×AceQ qPCR SYBR Green Master Mix (10 µL) (Vazyme), template cDNA (1 µL), each of forward and reverse primer (0.5 µL;10 µM), and ddH_2_O (8 µL). The PCR system was programmed as described by Liu et al. (2021b). Normalization was achieved using the wheat actin gene (GenBank: AB181991.1) as the internal standard (Dudziak et al., 2020; Liu et al., 2022), with relative expression levels determined by the 2^-ΔΔCT^ method (Livak and Schmittgen, 2001). For the qRT-PCR experiment, three replicates were established for each stage of the normal and HT-ms anther samples. The significance of differences was assessed using Student’s t-test in SPSS software version 27.0, with P ≤0.05, P ≤ 0.01, and P ≤ 0.001 indicating significant, extremely significant, and most significant differences, respectively.

## Results

### Phenotypic differences in anthers between the normal and HT-treated wheat

**Figure 1:**
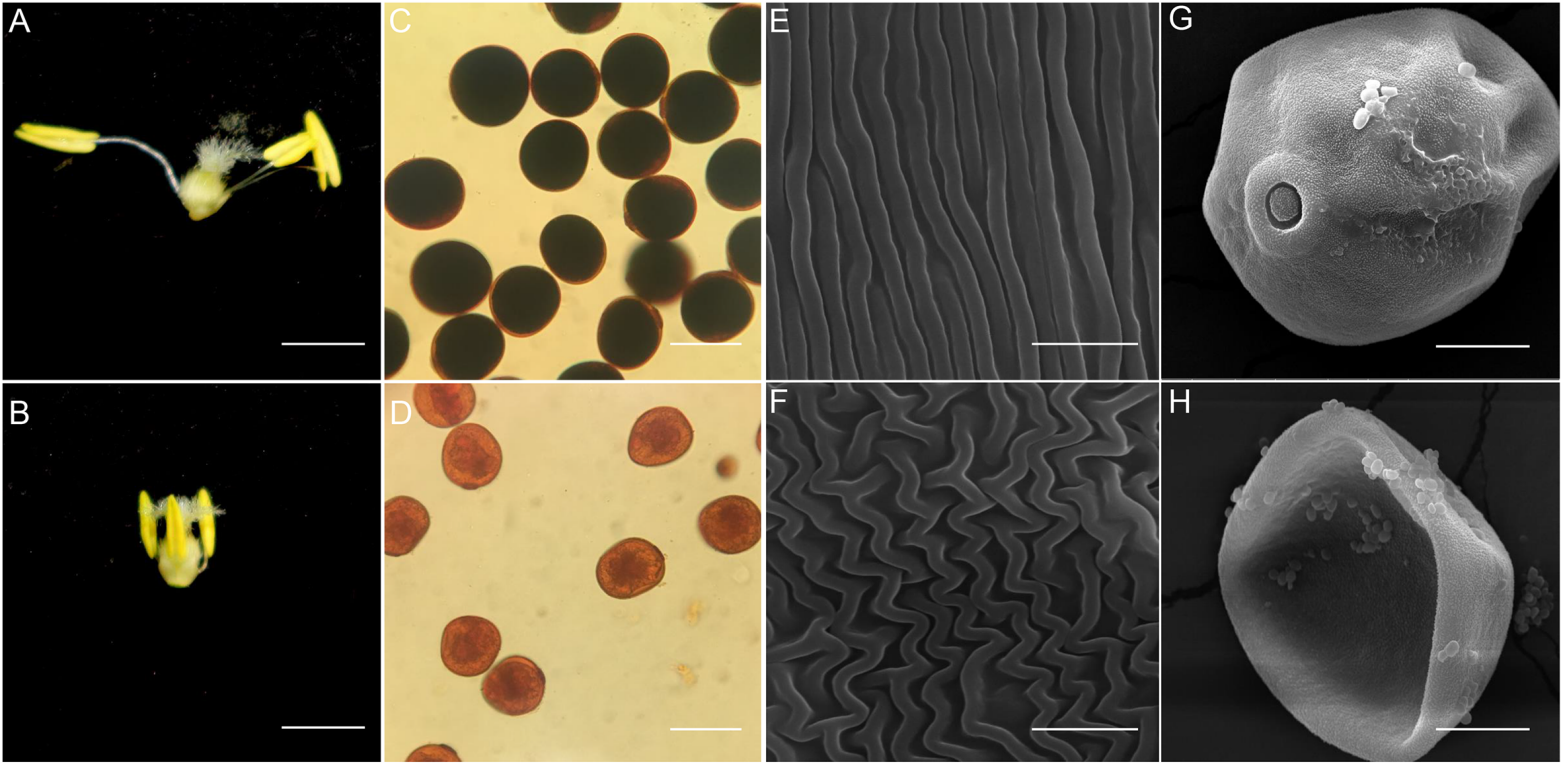
Appearance of the trinuclear stage anther, anther outer walls and pollen grain in normal and HT-ms plant. **(A, B)** The morphology of normal trinuclear stage anthers **(A)** and HT-ms trinuclear stage anthers **(B)**. **(C, D)** Staining results of pollen grains of normal **(C)** and HT-ms plants **(D)** with potassium iodide-iodine (KI-I2) solution. **(E, F)** Observation of electron scanning microscopy (SEM) of the outermost layer of normal **(E)** and HT-ms **(F)** anther epidermis. **(G, H)** Pollen grains of normal **(G)** and HT-ms **(H)** plants were observed by SEM. Bars = 2 mm in **(A, B)** 100 µm in **(C, D)** 5µm in **(E, F)** and 20 µm in **(G, H)**.

Two main differences were observed in wheat anthers at anthesis: normal wheat anthers were slightly larger than those subjected to HTs, and the anther filaments underwent elongation and dehiscence rapidly in normal wheat but not in wheat subjected to HTs (**Figures 1A, B**). We performed I_2_-KI solution staining to evaluate starch accumulation in the mature pollen grains and observed that normal pollen grains turned fully black, indicating complete starch accumulation (**Figure 1C**). By contrast, pollen grains from HT-ms anthers failed to appear black color after staining, indicating minimal or no starch accumulation (**Figure 1D**). Furthermore, we performed SEM to examine the ultrastructural characteristics of these anthers and pollen grains. The outer surface of normal anthers appeared flat and smooth, whereas that of HT-induced anthers was disordered (**Figures 1E, F**). The HT-induced anthers had severely malformed pollen shape, whereas the normal pollen grains exhibited a particulate exine pattern and a nearly round shape (**Figures 1G, H**). These SEM findings are consistent with those of I_2_-KI staining, indicating the defective development of the anthers and pollen grains in the HT-induced plants. Sectioned anthers from these stages were compared. The findings revealed that each wheat anther contains four symmetrical pollen capsules. At the tetrad stage, sterile anthers exhibited a thinner tapetum layer and deformed tetrad microspores compared with normal anthers (**Supplementary Figures S1A, B**). At the binuclear stage, normal anther microspores had accumulated starch and appeared full, although the internal material was not fully accumulated, whereas sterile anther microspores did not exhibit these characteristics (**Supplementary Figures S1C, D**). At the trinucleate stage, the epidermis of normal anthers was thinner with slits, containing round, mature pollen grains, whereas the epidermis of sterile anthers was thicker without slits, containing irregularly shaped pollen grains with little or no starch accumulation (**Supplementary Figures S1E, F**).

### Identification of proteins and functional annotation

To identify key proteins associated with the onset of HT-induced male sterility, we performed TMT proteomic analysis of wheat anthers at critical development stages: tetrad, binuclear, and trinuclear. We performed principal component analysis (PCA) and calculated the relative standard deviation values within each group and Pearson’s correlation coefficients. The findings revealed that the overall differences between the samples in each group and the variability within the group samples were small. These results indicated that the samples met the identification requirements and that the biological replicates within each group exhibited satisfactory reproducibility (**Supplementary Figures S2A–C**). We identified 9366 proteins, of which 8548 were annotated. In particular, 8186, 4609, and 4499 proteins were annotated in the GO, KEGG, and COG databases, respectively, and 2876 proteins were annotated across all the three databases (**Supplementary Figure S2D**).

### Identification of DAPs

To identify proteins associated with HT-induced male sterility during anther development, we performed TMT labeling of peptides for the global quantification of anther proteins across three developmental stages: tetrad, binuclear, and trinucleate. Through pairwise comparisons among the three developmental stages, we identified 2532 DAPs, with the tetrad, binuclear, and trinuclear stages having 27, 157, and 2348 DAPs, respectively. The detailed information about these DAPs is provided in **Supplementary Table S2**.

**Figure 2:**
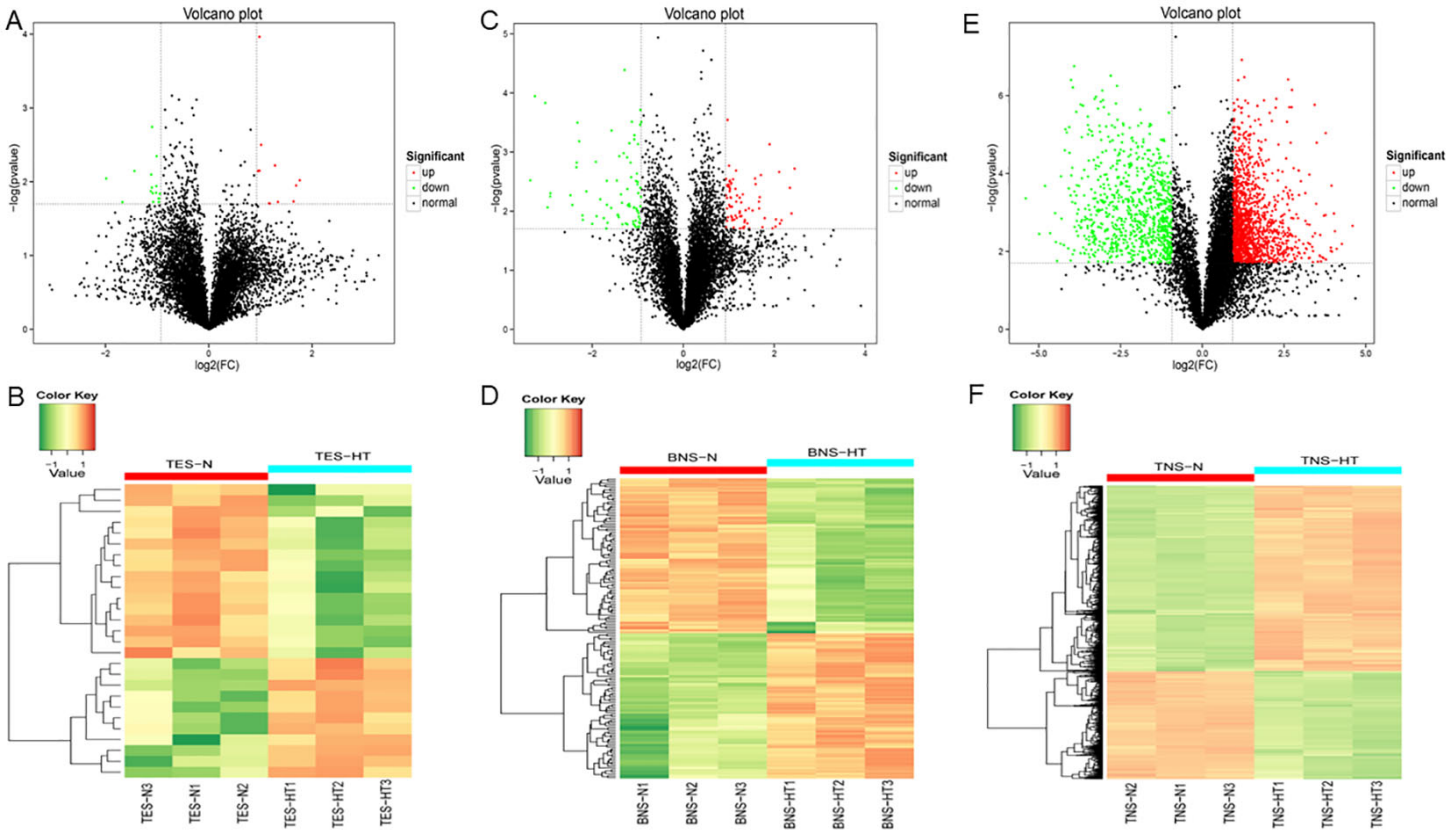
The volcano plot and clustering heat map show the changes in the abundance of differentially abundant proteins (DAPs) between fertile anthers and HT-ms anthers. **(A, B)** The volcano plot **(A)** and clustering heat map **(B)** of DAPs during the tetrad stage. **(C, D)** The volcano plot **(C)** and clustering heat map **(D)** of DAPs during the binuclear stage. **(E, F)** The volcano plot **(E)** and clustering heat map **(F)** of DAPs during the trinuclear stage. TES, tetrad stage. BNS, binuclear stage. TNS, trinuclear stage.

At the tetrad stage, volcano and heat maps revealed the upregulation of 11 DAPs, with the highest multiplicity and most significant upregulation displayed by an uncharacterized protein (TraesCS7B02G029200.1) and carboxypeptidase (TraesCS7A02G150900.1); these maps also revealed the downregulation of 16 proteins, with Formin-like protein (TraesCS1B02G094200.1) and an uncharacterized protein (TraesCS5A02G115300.2) exhibiting the most significant downregulation (**Figures 2A, B**). At the binuclear stage, out of 157 DAPs, 76 were upregulated and 81 were downregulated. The proteins with the largest FC increase were Hsp20 domain-containing protein (TraesCS4A02G298600.1) and shikimate dehydrogenase (NADP (+)) (TraesCS3B02G229000.1), whereas those with the largest FC decrease were pentatricopeptide repeat-containing protein MRL1 (TraesCS1A02G133500.4) and an uncharacterized protein (TraesCS2D02G393500.1; **Figures 2C, D**). The levels of DAPs substantially increased in the trinuclear stage compared with the previous two stages, with the number of upregulated and downregulated DAPs being 1483 and 865, respectively. The proteins with the largest FC increase and decrease in this stage were an uncharacterized protein (TraesCS5A02G441600.1) and diacylglycerol kinase (TraesCS7B02G293200.1), respectively, with the most significant DAPs being a protein kinase domain-containing protein (TraesCS4D02G046900.1) and pyruvate kinase (TraesCS1D02G215900.1; **Figures 2E, F**). A venn diagram analysis was conducted to explore the DAPs with altered abundance and thus determine the proteome dynamics in the three different developmental stages. The diagram illustrates one shared DAP between the tetrad stage and the binuclear stage and six shared DAPs between the tetrad stage and the trinuclear stage; the number of shared DAPs between the binuclear and the trinuclear stages was 56 (**Supplementary Figure S3**).

### Functional annotation of DAPs

**Figure 3:**
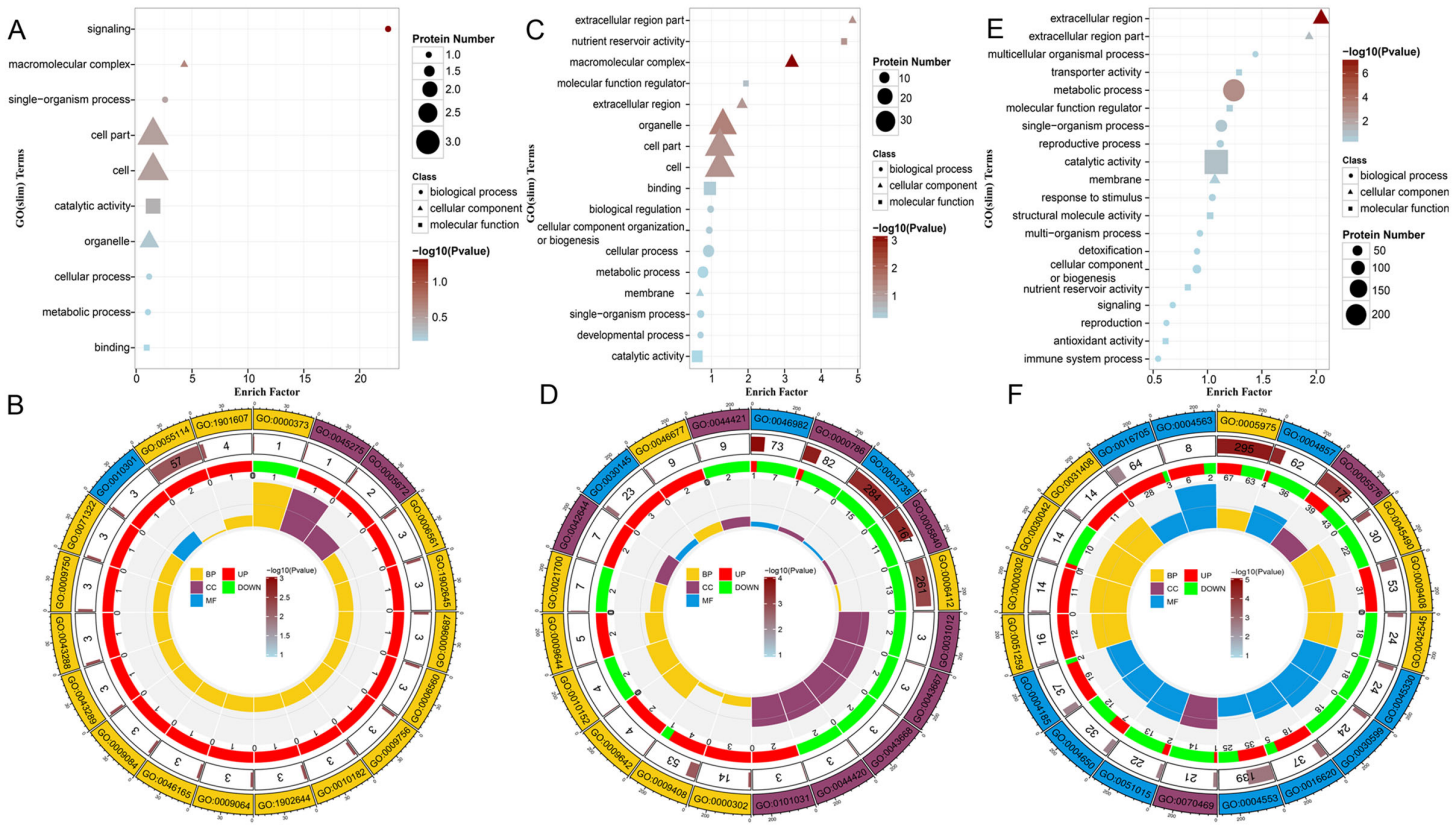
Presentative images showing top enrichment gene ontology (GO) terms of the differentially abundant proteins (DAPs). GOslim enrichment bubble plots **(A, C, E)** and GO enrichment circle plots **(B, D, F)** showing the enrichment of significant DAPs, where **(A, B)** is the tetrad stage, **(C, D)** is the binuclear stage, and **(E, F)** is the trinuclear stage.

To identify the critical pathways associated with the DAPs, we conducted GO enrichment and KEGG pathway analyses. For the tetrad stage, GO enrichment results indicated that biological processes were predominantly involved in signaling, single-organism processes, cellular processes, and metabolic processes. The cell components were enriched in cell, cell part, macromolecular complex, and organelle categories, whereas molecular functions were mainly enriched for catalytic activity and binding (**Figure 3A**). Furthermore, GO circle plots were used to identify pathways in which the DAPs were significantly enriched. The top five enriched biological processes were Group II intron splicing, oxidation-reduction process, proline biosynthetic process, tertiary alcohol biosynthetic process, and abscisic acid metabolic process. Notable cellular components included the transcription factor TFIIA complex and respiratory chain complex III, with xanthoxin dehydrogenase activity being a significant molecular function (**Figure 3B**). During the binuclear stage, GO enrichment analysis revealed enrichment of DAPs in biological processes related to cellular and metabolic processes, biological regulation, cellular component organization or biogenesis, single-organism processes, and developmental processes. (**Figure 3C**). Specific biological processes of interest included translation, response to reactive oxygen species (GO:0000302), and heat response (GO:0009408). Cellular components noted were pollen wall (GO:0043667), exine (GO:0043668), and extracellular matrix (GO:0031012), whereas the main molecular functions were manganese ion binding (GO:0030145), protein heterodimerization activity, and structural constituent of ribosome (**Figure 3D**). In the trinuclear stage, GO results indicated significant enrichment of DAPs in biological processes such as metabolic processes, single-organism processes, cellular component organization, reproductive processes, response to stimuli, signaling, and reproduction. (**Figure 3E**). The biological processes illustrated in the circle diagram of the trinuclear stage included carbohydrate metabolic processes (GO:0005975), pectin catabolic processes (GO:0045490), heat response (GO:0009408), cell wall modification (GO: 0042545), and response to ROS reaction (GO:0000302).Cellular component processes included the respiratory chain and extracellular region. Molecular functions included hydrolase activity, O-glycosyl compound hydrolysis, oxidoreductase activity, and others (**Figure 3F**). **Supplementary Tables S3**, **S4** present the details of the aforementioned GO enrichment analyses.

**Figure 4:**
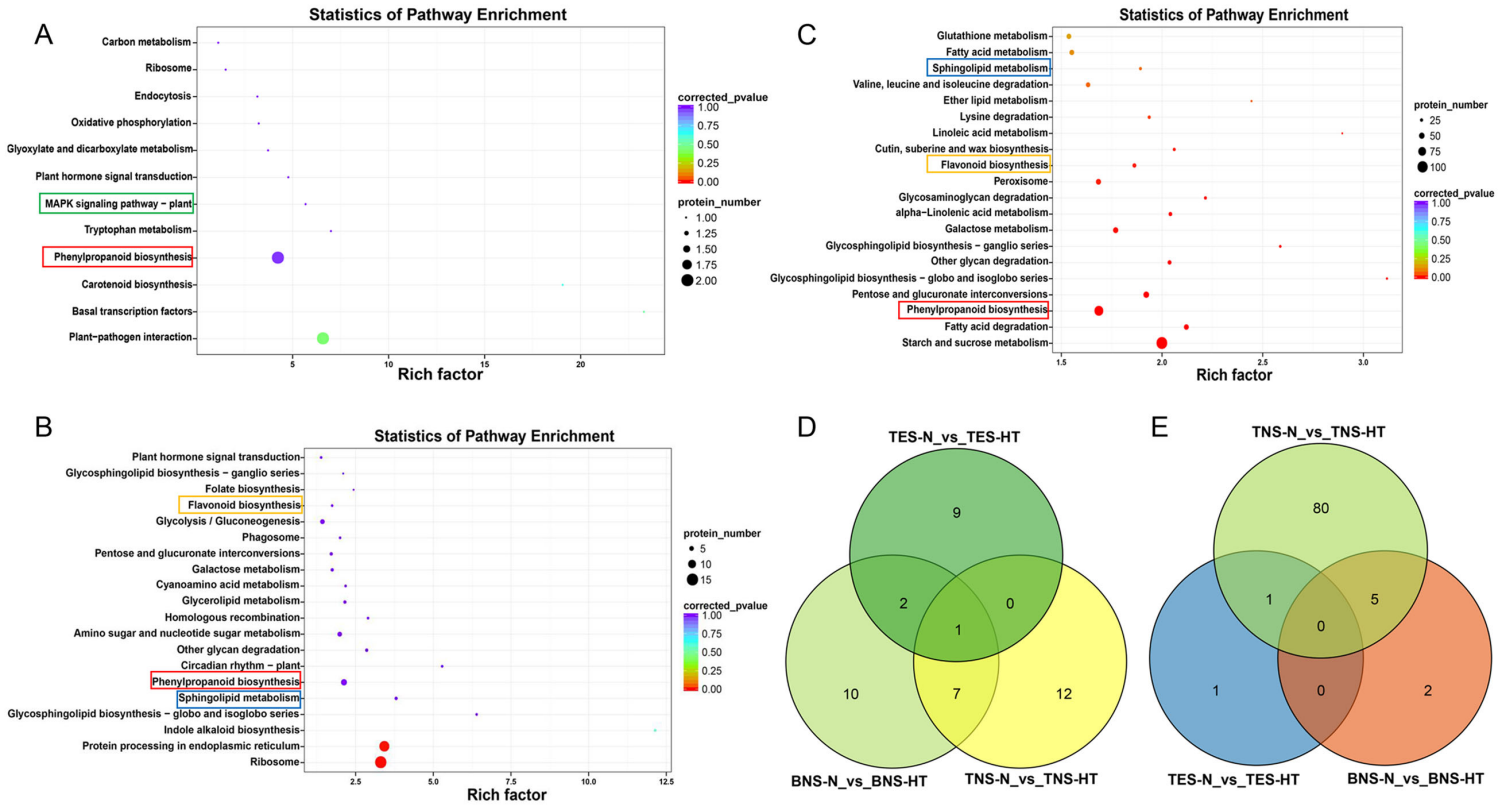
Scatter plot of KEGG pathway enrichment for differentially abundant proteins (DAPs). **(A–C)** Scatter plot for DAPs at the tetrad stage **(A)**, the binuclear stage **(B)**, and the trinuclear stage **(C)**. **(D)** Venn diagram results for the top 20 KEGG enrichment pathway at three developmental stages. **(E)** Venn diagram results of DAPs of the phenylpropanoid biosynthesis pathway at three developmental stages. Highlight pathways marked in different colors as the focal pathways of interest. TES, tetrad stage. BNS, binuclear stage. TNS, trinuclear stage.

The DAPs across the three developmental stages were annotated as 12 (tetrad), 50 (binuclear), and 50 (trinuclear) KEGG metabolic pathways, grouped into five categories: cellular processes, environmental information processing, genetic information processing, metabolism, and biological systems (**Supplementary Figure S4**). Notably, the KEGG pathway analysis during the anther development stages highlighted phenylpropanoid biosynthesis (Ko00940) as a common enriched pathway in the top 20 across all stages. The tetrad and binuclear stages shared two pathways: “ribosome (Ko03010)” and “plant hormone signal transduction (Ko04075).” Between the binuclear and trinuclear stages, seven shared pathways were identified: “flavonoid biosynthesis (Ko00941),” “sphingolipid metabolism (Ko00600),” “other glycan degradation (Ko00511),” “galactose metabolism (Ko00052),” “pentose and glucuronate interconversions (Ko00040),” “glycosphingolipid biosynthesis-ganglio series (Ko00604),” and “glycosphingolipid biosynthesis-globo and isoglobo series (Ko00603)” (**Figures 4A–D**). Furthermore, a Venn diagram demonstrated that gene counts in the phenylpropanoid biosynthesis pathway were 2, 7, and 86 for the tetrad, binuclear, and trinuclear stages, respectively, with one gene shared between the tetrad and trinuclear stages and five genes shared between the binuclear and trinuclear stages (**Figure 4E**). To determine the enrichment status of these DAPs, KEGG enrichment analysis was performed on the upregulated and downregulated proteins at each stage. The analysis indicated that the metabolic pathways significantly enriched for downregulated proteins were “phenylpropanoid biosynthesis,” “ribosome,” and “starch and sucrose metabolism (Ko00500)” for the tetrad, binuclear, and trinuclear stages, respectively (**Supplementary Figure S5**). In addition, several pathways outside the top 20 for the binuclear and trinuclear stages might be relevant for exploring HT-induced male sterility, including “Cutin, suberine and wax biosynthesis,” “Phenylalanine, tyrosine and tryptophan biosynthesis,” “MAPK signaling pathway plants,” “fatty acid degradation,” “isoflavonoid biosynthesis,” and “phosphatidylinositol signaling system” (**Supplementary Table S5**).

The analysis of subcellular localization for the DAPs across the three stages revealed that the nucleus, cytoplasm, and plasma membrane were the predominant locations, with the plasma membrane exhibiting the most significant DAP enrichment (**Supplementary Figure S6**). Moreover, when examining the protein family (PFAM) annotations and enrichment results, we found that the GCK domain had the highest enrichment factor during the tetrad stage. By contrast, the Hsp20/alpha crystalline family demonstrated the highest numbers and significance during the binuclear and trinuclear stages (**Supplementary Figure S7**).

**Figure 5:**
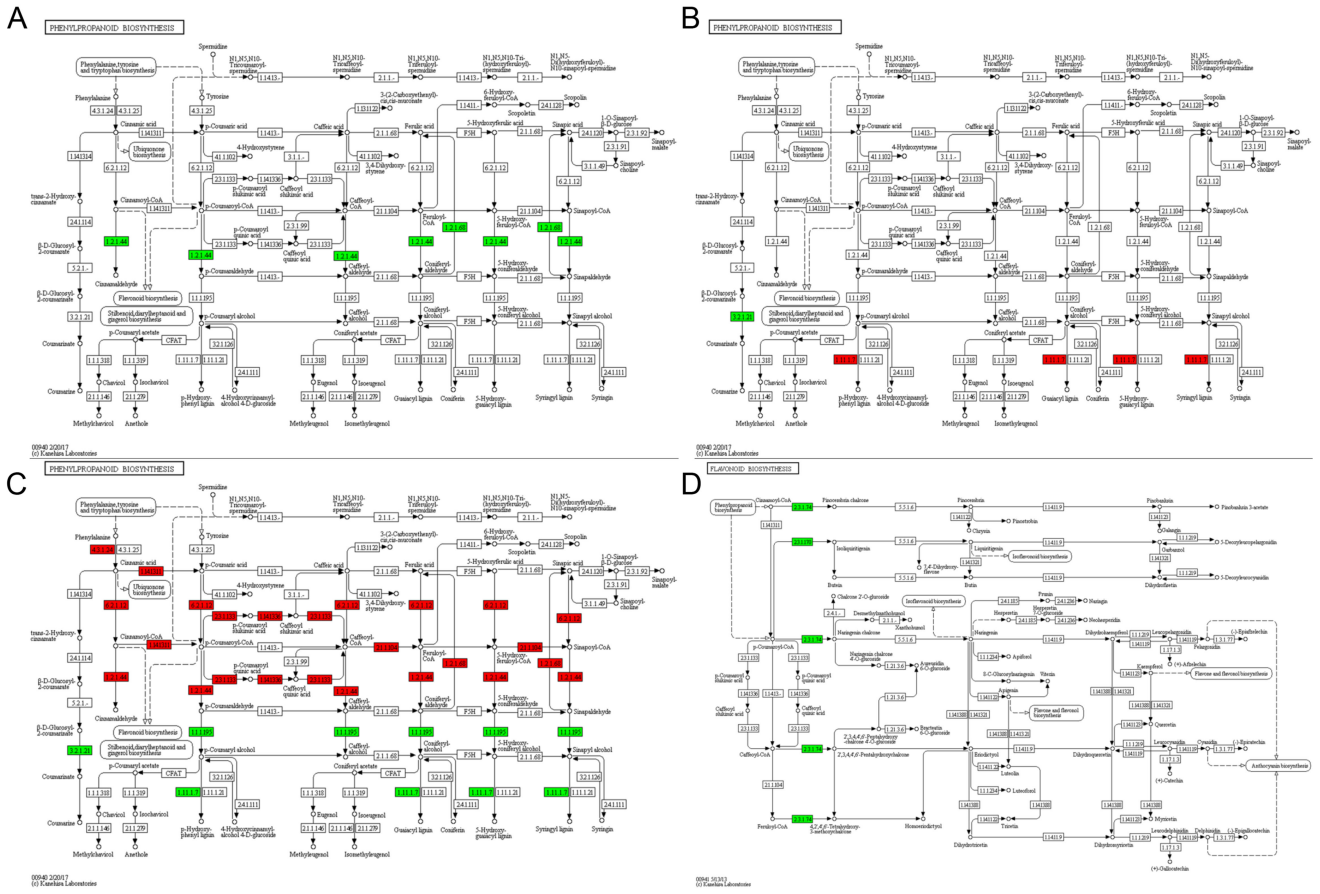
KEGG pathway map. Maps of the KEGG pathway for phenylpropanoid biosynthesis during the tetrad **(A)**, binuclear **(B)** and trinuclear **(C)** stages. Map of the KEGG pathway for flavonoid synthesis in the binuclear stage **(D)**.

Through the integration of GO and KEGG analyses, we focused on seven pivotal pathways: phenylpropanoid biosynthesis, flavonoid biosynthesis, sphingolipid metabolism, MAPK signaling pathway-plant, starch and sucrose metabolism, response to heat, and response to ROS. The KEGG enrichment map for phenylpropanoid biosynthesis indicated that genes coding for key enzymes in the lignin biosynthesis pathway, specifically cinnamoyl-CoA reductase (EC:1.2.1.44) and coniferyl-aldehyde dehydrogenase (EC:1.2.1.68), were downregulated in the tetrad stage of sterile anthers subjected to HT (**Figure 5A**). At the binuclear stage, a gene involved in the coumarinate production pathway, beta-glucosidase (EC:3.2.1.21), exhibited downregulation, whereas genes associated with peroxidase (EC:1.11.1.7) displayed upregulation in sterile anthers subjected to HT compared with normal anthers (**Figure 5B**). Furthermore, 29 genes involved in the lignin synthesis pathway’s downstream key enzymes—beta-glucosidase, peroxidase, and cinnamyl alcohol dehydrogenase (CAD, EC:1.1.1.195) demonstrated a decreased expression compared with normal anthers at the trinucleate stage (**Figure 5C**). In the binuclear stage, two proteins related to the flavonoid biosynthesis pathway, linked to chalcone synthase (EC:2.3.1.74), were downregulated in HT-ms anthers, marking a crucial step in the flavonoid metabolism pathway (**Figure 5D**). In addition, at the trinuclear stage, certain DAPs in HT-ms anthers were downregulated in key enzymes involved in the production pathways of flavonoid-related intermediates or end products at the trinuclear stage, such as, such as chalcone isomerase (EC:5.5.1.6) and flavonol synthase (EC:1.14.11.23) (**Supplementary Figure S8**).

**Figure 6:**
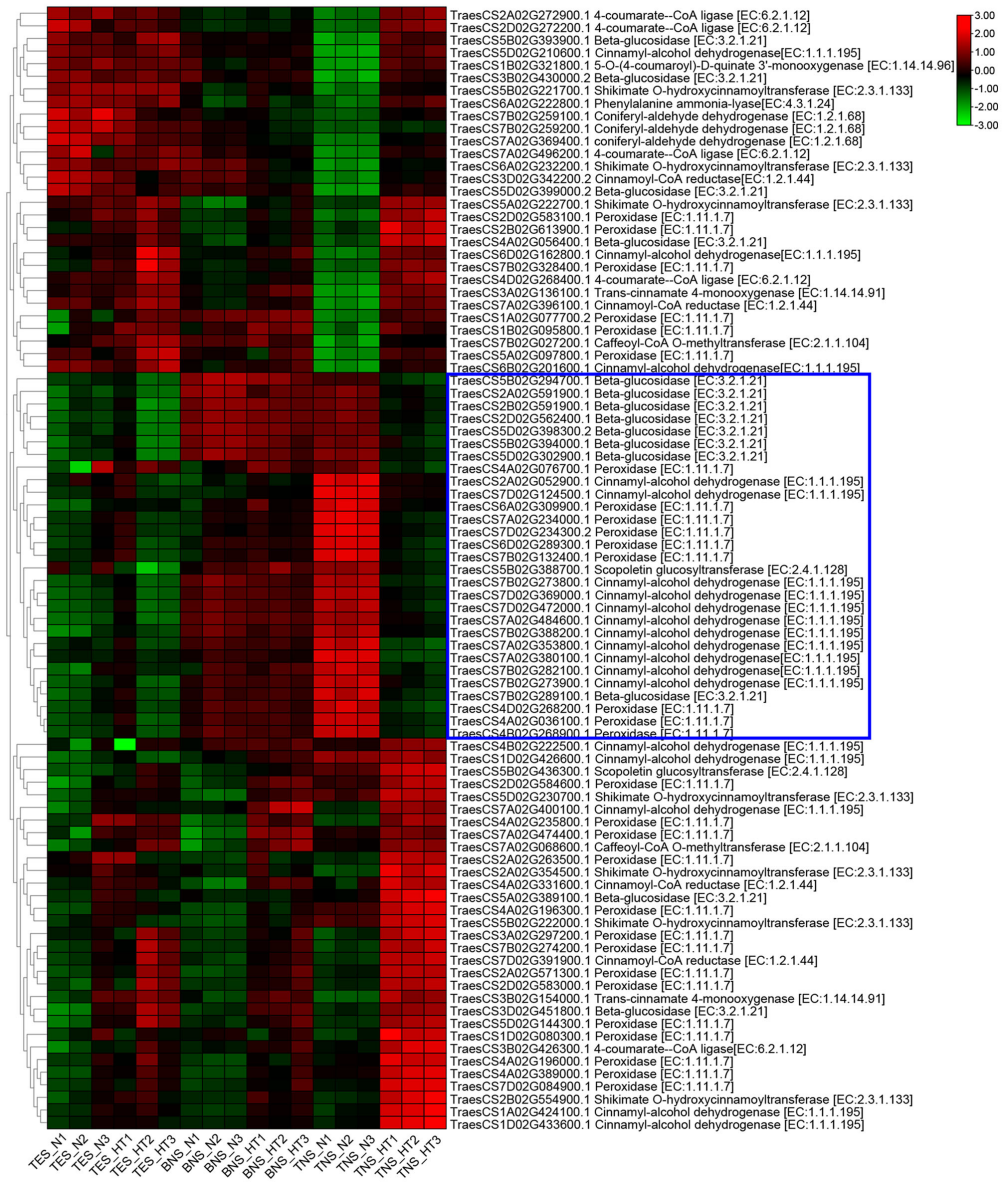
The heatmap of differentially abundant proteins (DAPs) involved in phenylpropanoid biosynthesis pathway. Blue boxes indicate DAPs that are significantly down-regulated for expression during the trinuclear stage. TES, tetrad stage. BNS, binuclear stage. TNS, trinuclear stage.

**Figure 7:**
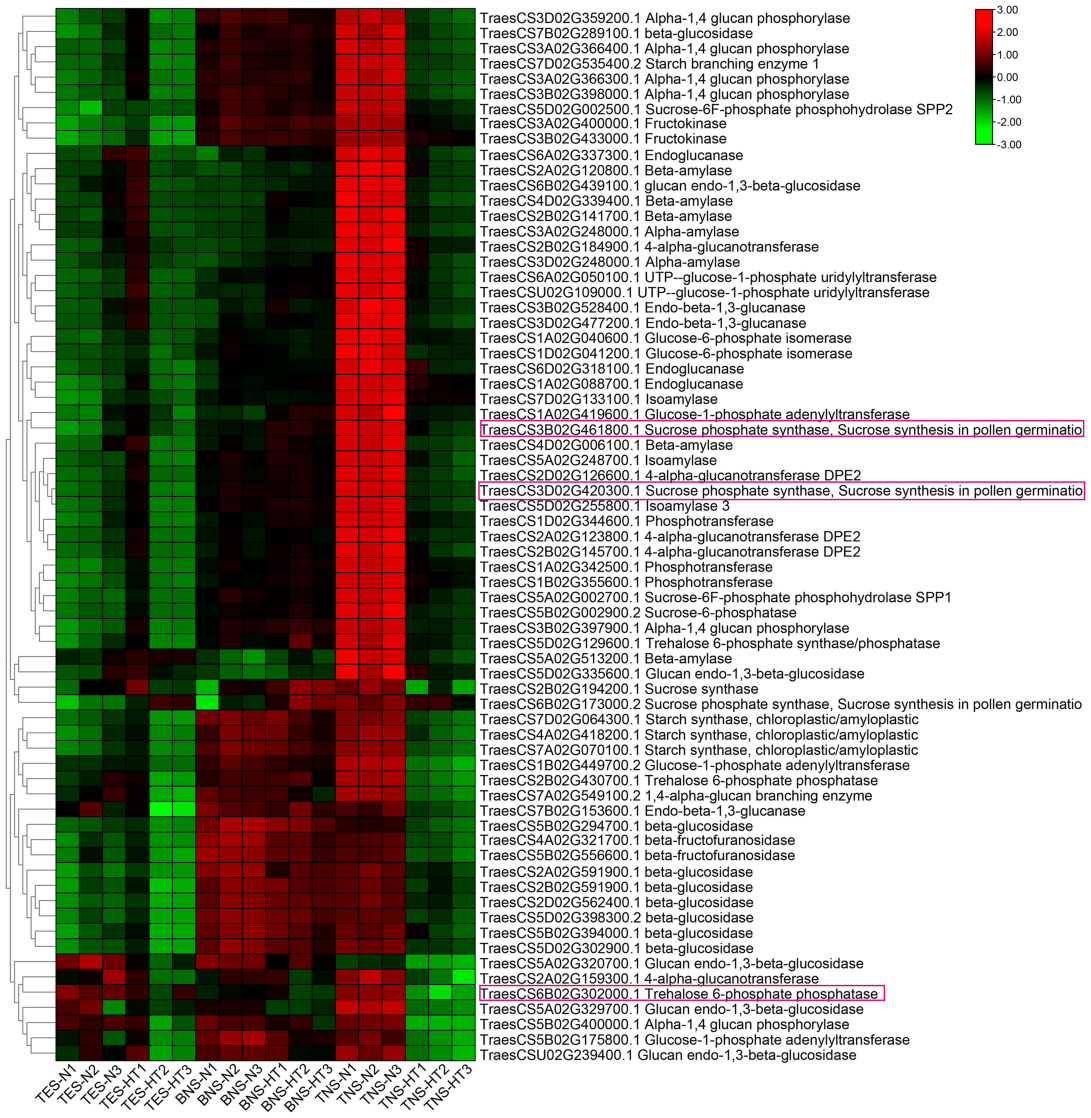
The heatmap for differentially abundant proteins (DAPs) in the starch and sucrose metabolic pathway. DAPs with purple boxes are the focal DAPs of interest. TES, tetrad stage. BNS, binuclear stage. TNS, trinuclear stage.

**Figure 8:**
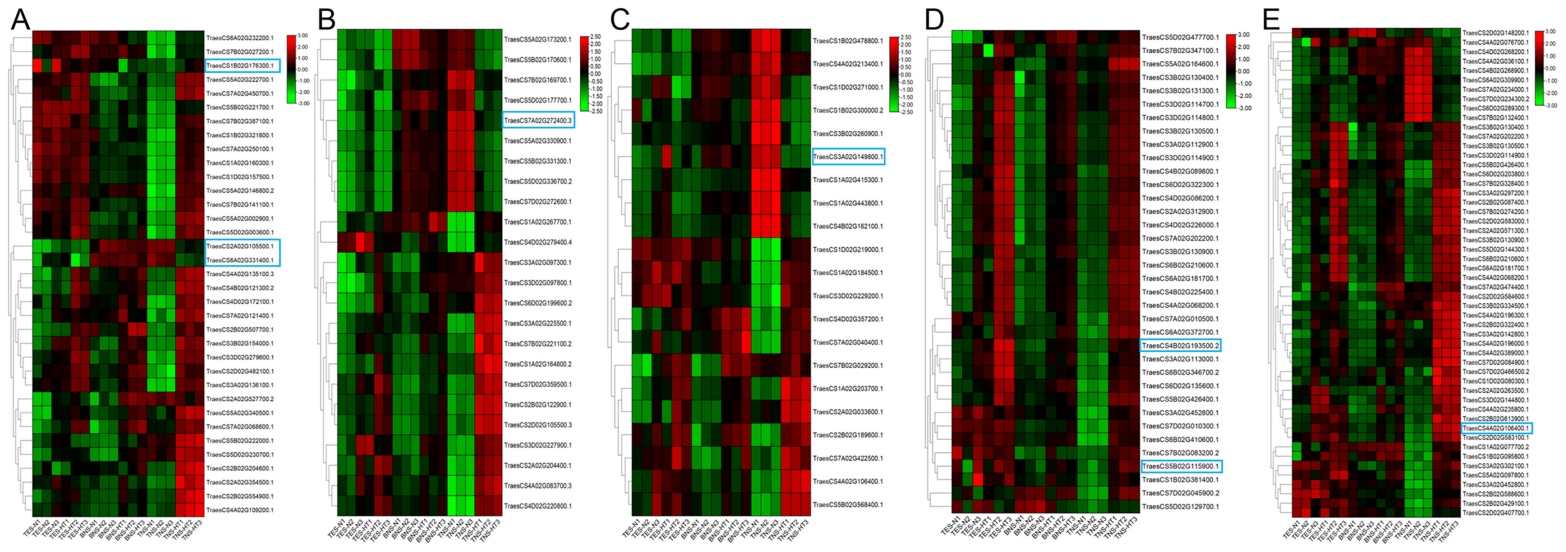
The heatmap for differentially abundant proteins (DAPs) in the other hub pathways. **(A–E)** The flavonoid biosynthesis pathway **(A)**, sphingolipid metabolism pathway **(B)**, MAPK signaling pathway **(C)**, heat stress-related pathways **(D)**, and ROS pathway **(E)**. TES, tetrad stage. BNS, binuclear stage. TNS, trinuclear stage.

We conducted a heatmap analysis of DAPs within the phenylpropane synthesis pathway. Our findings revealed that the DAPs downregulated at the trinuclear stage were primarily associated with beta-glucosidase, CAD, and peroxidase, aligning with our KEGG enrichment findings (**Figure 6**; **Supplementary Table S6**). The heatmap for DAPs in the starch and sucrose metabolic pathway indicated a uniform downregulation of DAP expression in sterile anthers at the trinuclear stage compared with normal anthers (**Figure 7**; **Supplementary Table S7**). In addition, the heatmap demonstrated significant downregulation at the binuclear stage of the DAP TraesCS1B02G176300.1 (chalcone synthesis) and at the trinuclear stage of the DAPs TraesCS2A02G105500.1 (chalcone isomerase) and TraesCS6A02G331400.1 (flavonol synthesis) in HT-ms anthers compared with normal anthers (**Figure 8A**; **Supplementary Table S8**). DAPs associated with the key enzymes involved in the sphingolipid metabolism pathway, including alpha-galactosidase and beta-galactosidase, were downregulated in HT-ms anthers at both the binuclear and trinuclear stages, as indicated by heatmap data for DAPs such as TraesCS5A02G173200.1 (**Figure 8B**; **Supplementary Table S9**). The MAPK signaling pathway DAPs exhibited varied expression, with both upregulation and downregulation observed in the heatmap (**Figure 8C**; **Supplementary Table S10**). The heat stress-related pathways “response to heat” and “response to temperature stimulus,” were upregulated in HT-ms anthers across all developmental stages in the heatmap (**Figure 8D**; **Supplementary Table S11**). Moreover, the heatmap analysis of DAPs associated with the ROS pathway revealed their increased expression during all developmental stages, particularly at the trinuclear stage, where 41 out of 52 genes were upregulated, accounting for 78.8% of the total (**Figure 8E**; **Supplementary Table S12**). The downregulation enrichment analysis at the trinuclear stage also demonstrated significant enrichment in the phosphatidylinositol signaling system, with the expression of 10 out of 13 DAPs being significantly decreased, including proteins related to phosphatidylinositol 4-kinase A (TraesCS4A02G268300.1) and 1-phosphatidylinositol-4-phosphate 5-kinase (PIP5K: TraesCS4D02G128000.2) (**Supplementary Figure S9**).

**Figure 9:**
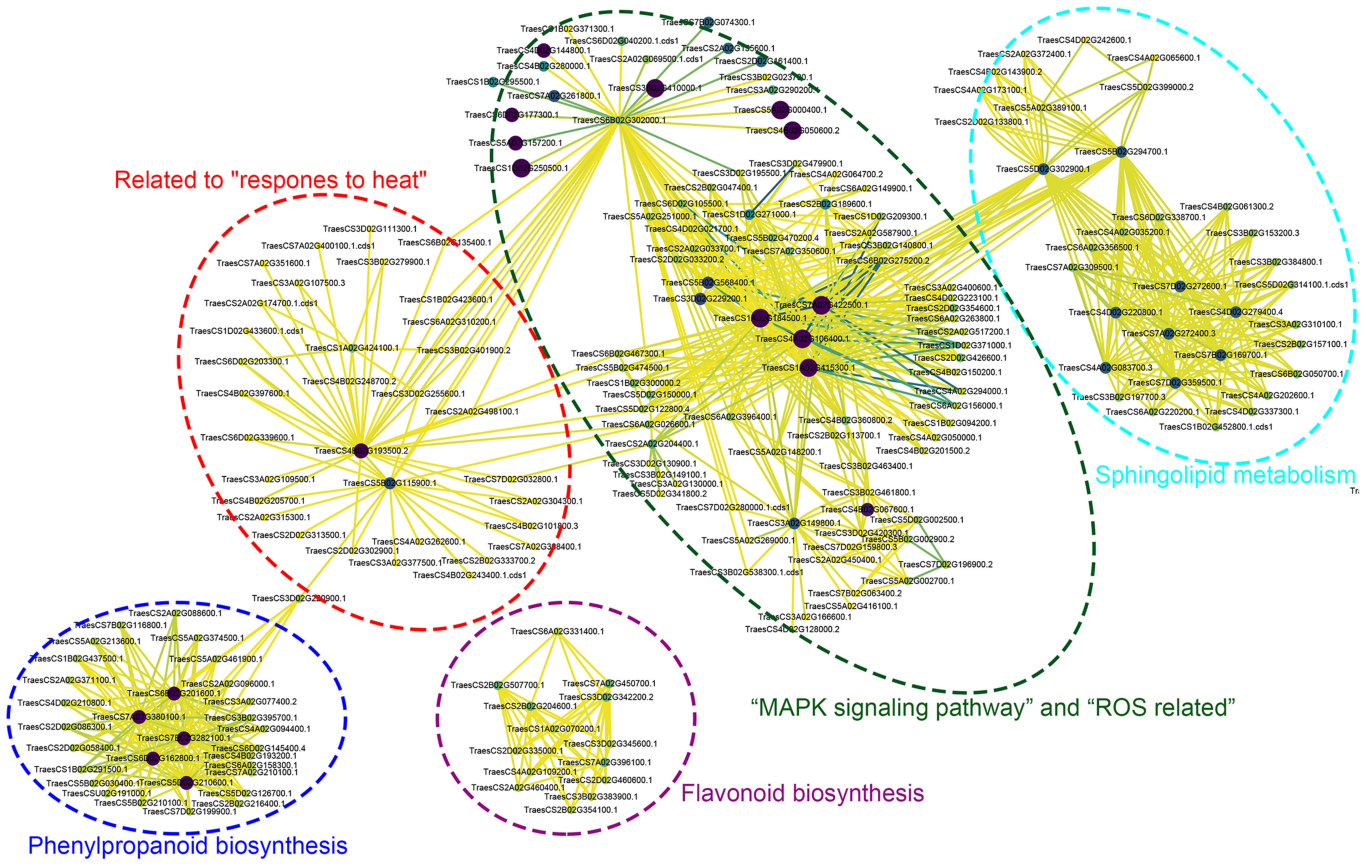
Protein-protein interaction (PPI) networks for critical pathways were mapped using STRING system and Cytoscape software. Select and represent the different involved pathways with dashed boxes of different colors. The detailed correspondence between transcript ID and the corresponding protein names is presented as follows: TraesCS7A02G380100.1 and TraesCS7B02G282100.1, cinnamyl-alcohol dehydrogenase; TraesCS7A02G272400.3, beta-galactosidase; TraesCS3A02G149800.1, dual specificity protein phosphatase 1B; TraesCS5B02G115900.1, Uncharacterized protein; TraesCS4A02G106400.1, mitogen-activated protein kinase 3; TraesCS6B02G302000.1, trehalose 6-phosphate phosphatase; TraesCS3D02G420300.1 and TraesCS3B02G461800.1, sucrose phosphate synthase.

To further identify central proteins within these seven pathways, we conducted a PPI analysis on the DAPs. The PPI analysis revealed that, apart from the flavonoid biosynthesis pathway, the other pathways interconnected through GTP-binding or chaperone proteins and various enzyme interactions were involved in glucose metabolism. Within the phenylpropanoid biosynthesis pathway, combining PPI results with KEGG-enriched downregulated protein analysis revealed TraesCS7A02G380100.1 and TraesCS7B02G282100.1 as key hub proteins. In the sphingolipid metabolism pathway, TraesCS7A02G272400.3 emerged as a central hub protein. Further analysis revealed the involvement of TraesCS7A02G272400.3 in sphingolipid metabolism, TraesCS3A02G149800.1 in the MAPK signaling pathway, TraesCS5B02G115900.1 in response to heat, and TraesCS4A02G106400.1 in the pathway related to ROS as hub proteins. For the starch and sucrose metabolism pathways, the identified hub proteins were TraesCS6B02G302000.1, TraesCS3D02G420300.1, and TraesCS3B02G461800.1 (**Figure 9**; **Supplementary Table S13**).

### Validation of the candidate hub proteins by qRT-PCR

**Figure 10:**
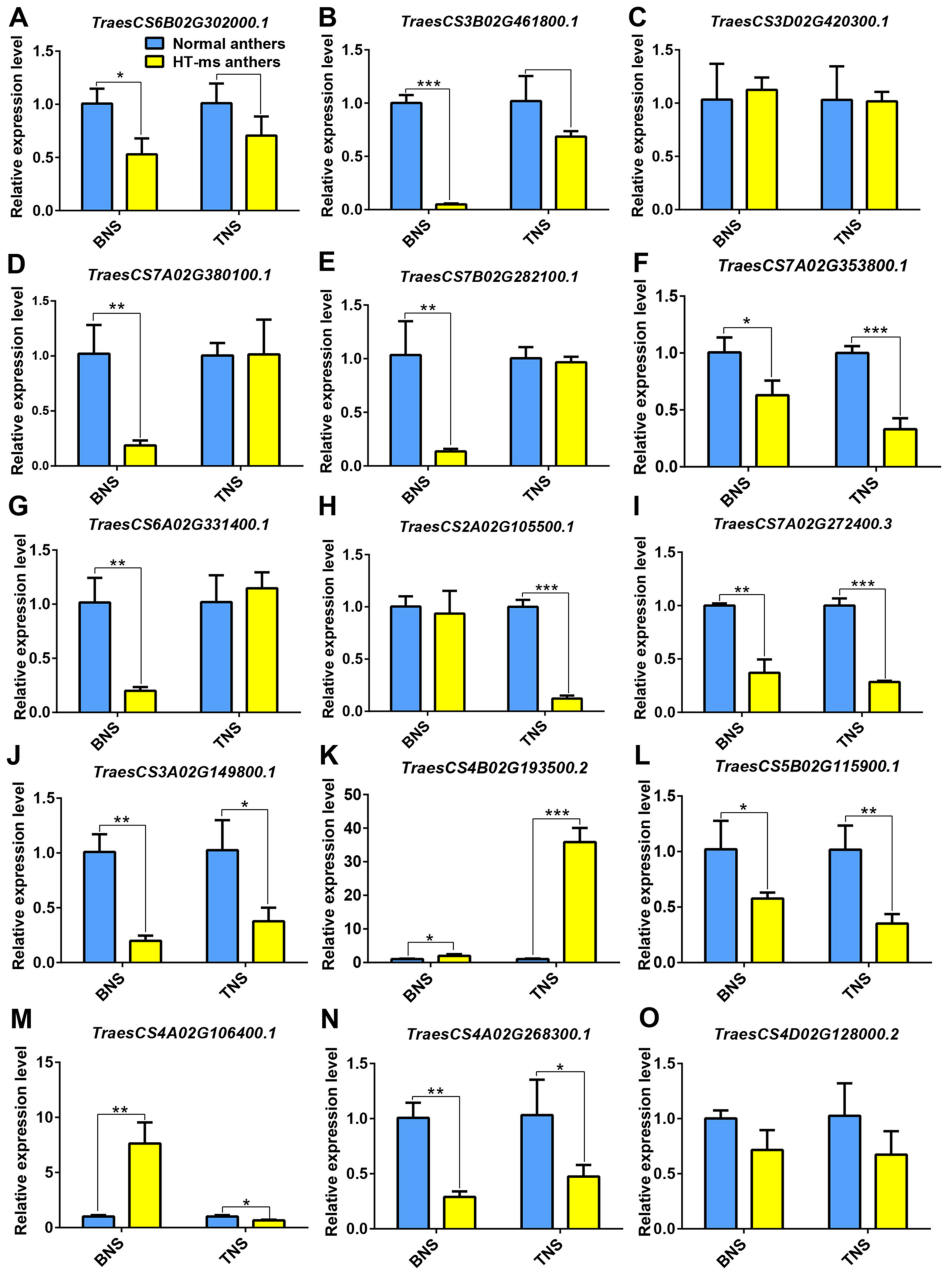
Differential expression of 15 genes of hub DAPs in normal and HT-ms anther tissues was analyzed by qRT-PCR. The developmental stages (binuclear and trinuclear stages) and relative expression levels of normal and HT-ms anthers are shown as the x-and y-axes, respectively. SPSS Statistics software and Graphpad Prism 6 project software was used to analyze the data as means of three replicates ± standard error. Capped lines indicate standard error. *P ≤ 0.05; **P ≤ 0.01, ***P ≤ 0.001. TES, tetrad stage. BNS, binuclear stage. TNS, trinuclear stage. The detailed correspondence between genes and the corresponding protein names is presented as follows: **(A)** TraesCS6B02G302000.1, trehalose 6-phosphate phosphatase; **(B, C)** TraesCS3B02G461800.1 and TraesCS3D02G420300.1, sucrose phosphate synthase; **(D–F)** TraesCS7A02G380100.1, TraesCS7B02G282100.1 and TraesCS7A02G353800.1, cinnamyl-alcohol dehydrogenase; **(G)** TraesCS6A02G331400.1, flavonol synthase; **(H)** TraesCS2A02G105500.1, chalcone isomerase; **(I)** TraesCS7A02G272400.3, beta-galactosidase; **(J)** TraesCS3A02G149800.1, dual specificity protein phosphatase 1B; **(K)** TraesCS4B02G193500.2, thermosensitive male sterile 1; **(L)** TraesCS5B02G115900.1, Uncharacterized protein; **(M)** TraesCS4A02G106400.1, mitogen-activated protein kinase 3; **(N)** TraesCS4A02G268300.1, phosphatidylinositol 4-kinase A; **(O)** TraesCS4D02G128000.2, 1-phosphatidylinositol-4-phosphate 5-kinase (**Supplementary Table S14**).

To validate the reliability of the DAPs and the abundance of hub proteins in the pathways associated with male sterility under HT stress, we selected 15 candidate DAPs for qPCR validation. The genes corresponding to the hub proteins of the starch and sucrose metabolism pathway, *TraesCS6B02G302000.1* and *TraesCS3B02G461800.1*, exhibited significant and extremely significant downregulation, respectively, at the binucleate stage in HT-ms anthers compared with normal anthers, with FCs of 1.90 and 20.45, respectively. At the trinucleate stage, their expression differences were not significant, but a clear downregulation trend was observed (**Figures 10A, B**). Another hub gene in this pathway, *TraesCS3D02G420300.1*, showed a slight upregulation at the binuclear stage and a mild downregulation at the trinucleate stage in HT-ms anthers, with the difference being nonsignificant (**Figure 10C**). Changes in the expression of these hub genes were consistent with the expression trends of the corresponding DAPs (**Figure 7**). For phenylpropanoid biosynthesis, all three genes exhibited significant downregulation in HT-ms anthers at the binuclear stage (**Figures 10D–F**). *TraesCS7A02G353800.1*, in particular, showed FCs of 0.60 and 2.02 at the binuclear and trinuclear stages, respectively, with the most significant difference observed at the trinuclear stage (**Figure 10F**). In flavonoid biosynthesis, the expression of *TraesCS6A02G331400.1* was significantly decreased at the binuclear stage and slightly increased at the trinuclear stage in HT-ms anthers, but without a significant difference. The other hub gene, *TraesCS2A02G105500.1*, showed a decreased expression at both stages, with an 8.20-fold decrease at the trinuclear stage, marking the most significant level of difference (**Figures 10G, H**). The sphingolipid metabolism hub gene, *TraesCS7A02G272400.3*, demonstrated highly significant downregulation at both stages in HT-ms anthers compared with normal anthers, with FCs of 1.70 and 2.52, respectively (**Figure 10I**). *TraesCS3A02G149800.1*, related to the MAPK pathway, showed significant downregulation at both stages, with FCs of 4.10 and 1.72, respectively (**Figure 10J**). The heat stress pathway gene, *TraesCS4B02G193500.2*, exhibited an upregulation trend, with fold increases of 1.99 and 35.53 at the binuclear and trinuclear stages, respectively, reaching significant and highly significant levels (**Figure 10K**). Conversely, the gene *TraesCS5B02G115900.1* showed a decreased expression at both binuclear and trinuclear stages, with extremely significant differences (**Figure 10L**). For the ROS-related pathway, the expression of *TraesCS4A02G106400.1* was 7.60-fold higher in binuclear HT-ms anthers than in normal anthers, with an extremely significant difference, whereas it showed a downregulation trend at the trinuclear stage with a significant difference (**Figure 10M**). Interestingly, the expression of the corresponding genes for the two hub proteins in the phosphatidylinositol pathway exhibited remarkable changes; TraesCS4A02G268300.1 exhibited extremely significant downregulation at both stages, with FCs of 2.47 and 1.17, respectively (**Figure 10N**), whereas the other gene, *TraesCS4D02G128000.2*, showed a decreasing expression trend at both stages but without significant differences (**Figure 10O**). Detailed qRT-PCR data are provided in **Supplementary Table S14**.

## Discussion

Global warming presents a significant challenge for humanity, affecting plants and global food production substantially. Reproductive processes in plants are particularly sensitive to rising temperatures, which can lead to male sterility in crops. The mechanisms by which HTs affect male sterility during reproductive development remain to be completely elucidated. In this study, using TMT proteomics, we explored the critical pathways and hub proteins associated with HT-induced male sterility in wheat. When anthers and pollen grains from HT-ms and normal plants were stained with I2-KI, the HT-ms samples showed little or no staining, indicating a lack of starch in the pollen grains, which is a marker of male sterility. GO enrichment analysis of the DAPs at the trinuclear stage revealed 130 DAPs associated with the carbohydrate metabolic process (GO:0005975) pathway, with 63 of them being downregulated. Additionally, 69 DAPs were found to be downregulated in the starch and sucrose metabolism pathway (Ko00500) in the KEGG analysis. This KEGG pathway analysis revealed a downregulation trend in several key enzymes, such as sucrose-phosphate synthase (EC: 2.4.1.14), sucrose-phosphate phosphatase (EC:3.1.3.24), UTP-glucose-1-phosphate uridylyltransferase (EC:2.7.7.9), NDP-glucose-starch glucosyltransferase (EC:2.4.1.242), isoamylase (EC:3.2.1.68), 1,4-alpha-glucan branching enzyme (EC:2.4.1.18), glycogen phosphorylase (EC:2.4.1.1), 4-alpha-glucanotransferase (EC: 2.4.1.25), and glucose-6-phosphate isomerase (EC:5.3.1.9), indicating a broad impact on this metabolic pathway (**Supplementary Figure S10**). The qRT-PCR validation of the genes corresponding to the hub proteins (TraesCS6B02G302000.1, trehalose 6-phosphate phosphatase; TraesCS3B02G461800.1 and TraesCS3D02G420300.1, sucrose phosphate synthase) of this pathway confirmed their significant downregulation (**Figures 10A–C**), aligning with the observed lack of starch accumulation in sterile anthers (**Figure 1D**). The relevance of the starch and sucrose metabolism pathway to male sterility is supported by studies in other plants. In wheat, differentially expressed genes in male-sterile lines were linked to hormone signal transduction and starch and sucrose metabolism (Zhang et al., 2022). Similar findings were reported in rice, where sugar and starch metabolic pathways are crucial for pollen fertility (Lee et al., 2022). In potatoes, proteomic analyses highlighted the significant role of starch and sucrose metabolism in pollen development (Huang et al., 2022), and similar associations were found in *Capsicum annuum*, where DAPs were associated with the starch, sucrose metabolism, and monoterpene biosynthesis pathways (Pei et al., 2022). These studies collectively affirm the close association of starch and sucrose metabolic pathways with male sterility.

The phenylpropanoid synthesis pathway, significantly enriched across all three phases in this study, plays a crucial role in the production of various secondary metabolites in land plants, including lignin, flavonoids, sporopollenin, lignans, hydroxycinnamic acid amides, and phenylpropanoid esters (Dong and Lin, 2021). This metabolic pathway, along with the timely degradation of the chorionic layer, is vital for the development of anthers and pollen. Lignin, a key end-product of the phenylpropanoid pathway, is essential for anther development. The process of lignification and the thickening of the anther endodermis are closely associated with anther dehiscence, affecting anther fertility either directly or indirectly (Gui et al., 2019). Transcriptomic analyses in photosensitive and thermosensitive genic male sterility (PTGMS) rice have highlighted that differentially expressed genes in these contexts are predominantly involved in the metabolic pathways of sugar, lipid, and phenylpropanoid (Sun et al., 2022). Research on the rice male-sterile mutant OsCCRL1, in which the activity of cinnamoyl-CoA reductases is low, revealed less lignin accumulation, disrupted phenylalanine metabolism, and delayed tapetum degradation (Zhang et al., 2023). Similarly, our study identified a downregulation trend in the DAPs related to the phenylpropanoid metabolic pathway, especially at the tetrad and trinuclear stages (**Figure 6**), suggesting a critical role of this pathway in HT-induced sterility in wheat, potentially influencing anther dehiscence (**Supplementary Figures S1E, F**). Cinnamoyl-CoA reductases (CCR, EC:1.2.1.44) catalyze the conversion of various hydroxycinnamoyl CoA esters, including cinnamoyl-coenzyme A, p-coumaroyl-coenzyme A, caffeoyl-coenzyme A, feruloyl-coenzyme A, and erucoyl-coenzyme A, to their corresponding aldehydes, providing precursors for lignin formation (Sonawane et al., 2013), and are considered key enzymes in lignin biosynthesis. Our findings indicated a decreased expression of CCR in HT-ms anthers at the tetrad stage, with TraesCS3D02G342200.2 (CCR) identified as a related DAP. Additionally, the KEGG pathway analysis showed a downregulation trend for coniferyl-aldehyde dehydrogenase (EC:1.2.1.68) during this phase, with TraesCS7B02G259200.1 (coniferyl-aldehyde dehydrogenase) as the associated DAP, suggesting that lignin synthesis is potentially compromised as early as the tetrad stage in HT-ms anthers.

Cinnamyl alcohol dehydrogenase (CAD) is a vital enzyme in the lignin biosynthesis pathway, catalyzing the final step in lignin biosynthesis (Preisner et al., 2018). Two DAPs (TraesCS7A02G380100.1, CAD; TraesCS7B02G282100.1, CAD) are significantly downregulated in all lignin monomer synthesis pathways. Consistently, in this study, the phenylpropanoid metabolism pathway’s KEGG map at the trinuclear stage revealed significant downregulation of two DAPs, TraesCS7A02G380100.1 and TraesCS7B02G282100.1, across all lignin monomer synthesis pathways. These DAPs, identified as CAD, were also identified as central hub proteins in the PPI analysis of the phenylpropanoid pathway, indicating a severe impact on lignin monomer synthesis. In this study, 11 DAPs annotated as CAD were found to be downregulated in the KEGG enrichment analysis for the trinuclear stage (**Figure 6**), implying a strong correlation between their expression and lignin synthesis. To validate these findings, qRT-PCR was conducted on hub genes associated with the phenylpropanoid pathway. The observed downregulation in HT-ms anthers aligns with the TMT proteomic analysis results (**Figures 10D–F**). In another context, the *Arabidopsis* irregular xylem8 mutants exhibit male sterility due to anther indehiscence, linked to reduced deposition of xylan and lignin in the endodermal cell layer (Hao et al., 2014). Similarly, in this study, a notable characteristic of HT-ms anthers was the lack of anther dehiscence, aligning with the results of the lignin synthesis pathway analysis. These results collectively indicate that the inhibition of lignin synthesis is a critical factor contributing to male sterility under HT conditions.

The biosynthesis of flavonoids is initiated through the phenylpropanoid pathway (Dong and Lin, 2021), where naringenin chalcone, a precursor in flavonoid synthesis, is produced from phenylalanine through the catalytic action of chalcone synthase on p-coumaroyl-CoA and malonyl-CoA (Yu et al., 2015). This chalcone is then converted into naringin by chalcone isomerase (Cheng et al., 2011). Flavonoids are crucial secondary metabolites that are involved in flower pigmentation, pollen fertility, and plant pollen exine formation (Liu et al., 2023). In *Arabidopsis*, the LESS ADHESIVE POLLEN (*LAP5*) and *LAP6* genes, which encode proteins similar to chalcone synthase and are specific to anthers, contribute to sporopollenin biosynthesis. They function in tandem with acyl-CoA synthetase 5, and mutations in these genes lead to irregular pollen wall morphology and male sterility (Dobritsa et al., 2010; Kim et al., 2010). In our current study, key enzymes of the flavonoid synthesis pathway, such as chalcone synthase, chalcone isomerase, and flavonol synthase, were down-regulated in KEGG maps in both binuclear and trinuclear anthers. This indicates that flavonoids such as galangin, kaempferol, quercetin, naringenin, etc. are blocked in the synthesis pathway and their content may be reduced, which in turn affects the process of sporopollenin biosynthesis and leads to sterility. The results of qRT-PCR analyses of genes corresponding to down-regulated and enriched DAPs in the trinuclear stage confirmed that the down-regulated expression of the genes was consistent with the results of the DAPs (**Figures 10G, H**).

Sphingolipids are crucial elements of lipid rafts within eukaryotic cell membranes, playing roles in growth and development regulation and cellular signal transduction. The phosphorylation of sphingosine, catalyzed by sphingosine kinase, is implicated in various plant developmental processes and adaptation mechanisms to adversity stress (Lynch and Dunn, 2004). Additionally, the homeostasis of sphingolipids is linked to PCD regulation, response to abscisic acid, and pollen fertility (Coursol et al., 2003; Wu S. et al., 2019). In this study, the expression of 24 DAPs associated with sphingolipid metabolism was most notably altered during the trinuclear stage, with 9 DAPs showing downregulation, including a key protein in this pathway (TraesCS7A02G272400.3, beta-galactosidase), and 15 DAPs exhibiting upregulation. This pattern suggests that proteins involved in sphingolipid metabolism may have an indirect impact on pollen fertility under HT stress (**Figure 8B**).

Our previous transcriptomic analyses indicated an accumulation of ROS in HT-ms anthers (Liu et al., 2021a). This finding is supported by the current proteomic study results, which identified numerous DAPs associated with ROS (**Figures 8E**, **9**). PPI analysis revealed that these DAPs are connected or exhibit interactions with proteins related to starch and sucrose metabolism and the MAPK signaling pathway. Notably, the protein TraesCS4A02G106400.1 (mitogen-activated protein kinase 3) is involved in both the ROS-related pathway and the MAPK pathway, highlighting its role in pollen development (**Figure 9**). Research on cytoplasmic male-sterile lines suggests that the overaccumulation of ROS, acting as a signal, may correlate with increased enzyme gene expression, disrupting the balance of the antioxidant system. This imbalance can lead to a delay in the initiation of tapetal PCD, resulting in male sterility (Liu et al., 2018). The results from the TMT proteomic analysis of thermosensitive sterile lines further corroborate these findings, demonstrating a notable decrease in soluble sugar and ATP contents, along with an abnormal accumulation of ROS (Ma et al., 2022).

These findings indicate the critical role of ROS balance in maintaining pollen fertility and highlight the complex interplay of various metabolic pathways in the process of anther development and male sterility. Our findings revealed a significant association between male sterility development and ROS accumulation, aligning with other research outcomes. The MAPK signaling pathway is crucial for cellular responses, transmitting external signals to the nucleus, and is conserved across species. MAPK enzymes, activated by ROS, play a key role in regulating the heat stress response in wheat under such conditions (Kumar et al., 2020). In our study, the MAPK pathway was identified as having DAPs with both increased and decreased expression, where TraesCS3A02G149800.1(dual specificity protein phosphatase 1B), a downregulated protein, had a hub position in the PPI analysis (**Figure 9**). qRT-PCR results also indicated a downregulation trend for the gene corresponding to this protein in HT-ms anthers at the trinuclear stage (**Figure 10J**), suggesting that the MAPK signaling pathway affects HT-induced male sterility in wheat, potentially interacting with ROS. The PPI analysis revealed a connection between the phenylpropanoid synthesis pathway, the MAPK pathway, and the ROS-related pathway through the “response to heat” (**Figure 9**). Heatmaps displaying the “response to heat” pathway proteins demonstrated that DAPs across all three stages were upregulated in sterile anthers (**Figure 8D**). This is further corroborated by qRT-PCR analysis of the hub protein-related genes, such as *TraesCS4B02G193500.2* (thermosensitive male sterile 1), which confirmed an upregulation in gene expression (**Figure 10K**). Conversely, the gene *TraesCS5B02G115900.1* (uncharacterized protein), associated with another hub protein, exhibited a different expression trend in qRT-PCR validation compared with the trend observed for proteins, which might be attributed to posttranscriptional and posttranslational modifications (**Figure 10L**). These findings highlight that a complex network of signaling pathways is involved in the development of male sterility under HT stress.

Our previous study revealed a connection between HT-ms anthers and PIP5K at the transcriptomic level (Liu et al., 2021b). In this study, KEGG enrichment analysis of downregulated proteins indicated significant enrichment of the phosphatidylinositol signaling system in HT-ms anthers at the trinuclear stage, with 10 out of 13 proteins in this pathway showing downregulation. The qRT-PCR analysis results for phosphatidylinositol 4-kinase A and PIP5K are in line with these proteomic findings, confirming their downregulation (**Figures 10N, O**). In pepper (*Capsicum annuum L.*), a comparative transcriptome analysis between sterile and fertility-restoring flower buds revealed *PIP5K* as a crucial gene for fertility restoration. Moreover, the phosphatidylinositol signaling system and phosphatidylinositol metabolism pathways were considered as the pathways most likely to affect fertility restoration (Wei et al., 2019). Similarly, in *Arabidopsis*, *PIP5K* and the phosphatidylinositol signaling pathway play essential roles during the early developmental stages of male gametogenesis (Ugalde et al., 2016). These insights collectively highlight the importance of PIP5K and the phosphatidylinositol signaling system in the regulation of anther male sterility, suggesting that disruptions in this signaling pathway could be a key factor for the development of sterility under HT conditions.

We infer that upon exposure to HT stress, wheat activates “response to heat” proteins, the MAPK pathway, and the phosphatidylinositol signaling system. However, this is accompanied by an increasing accumulation of ROS, leading to an imbalance. This imbalance has dual implications: it obstructs the starch and sucrose synthesis pathway, resulting in a lack of starch in sterile anthers, and it disrupts the normal function of the phenylpropanoid biosynthesis pathway, which affects the synthesis or the availability of key components, such as lignin and flavonoids, that are essential for anther dehiscence, thereby affecting fertility. In-depth research is needed to understand the intricate interplay between these pathways and the subsequent abnormal gene or protein expressions related to the phenylpropanoid metabolic pathway, resulting in HT-induced male sterility in wheat.

## Conclusion

This study uncovered the molecular mechanisms underlying male sterility induced by HTs in wheat at the proteomic level through a comparative proteomic analysis conducted using TMT-based proteomics technology, focusing on the differences between the normal and HT-ms anthers. A total of 2532 DAPs were identified across the tetrad, binucleate, and trinucleate stages of wheat anther development. Furthermore, we found that the phenylpropanoid biosynthesis pathway, the starch and sucrose metabolism pathway, and the accumulation of ROS are pivotal factors for the disruption of wheat anther dehiscence and the inhibition of starch accumulation, ultimately contributing to male sterility. This study sheds light on the molecular underpinnings of HT-induced male sterility in wheat and serves as a valuable resource for further research on plant sexual reproduction and the impact of environmental stress factors.

## Data Availability

The original contributions presented in the study are included in the article/**Supplementary Material**, further inquiries can be directed to the corresponding author/s.
