# Supplementary material for: Quantitative proteomic analysis reveals hub proteins for high temperature-induced male sterility in bread wheat (Triticum aestivum L.)
Source: Front Plant Sci. 2024 Sep 3;15:1426832. doi: 10.3389/fpls.2024.1426832 (PMC11405254; doi:10.3389/fpls.2024.1426832)
Supplement: Supplementary file 2 [file Presentation1.pptx]

## Slide 1
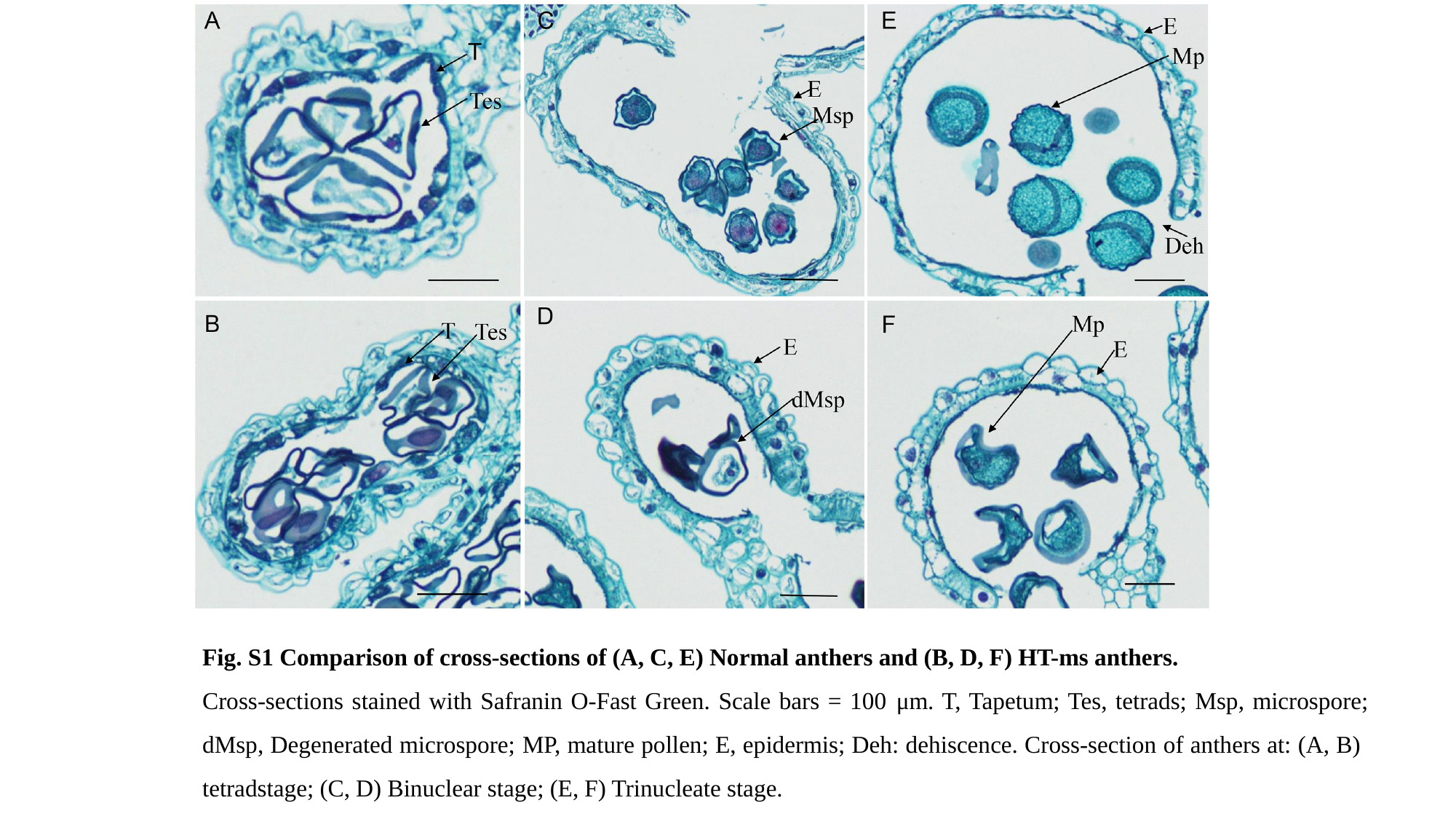

Fig. S1 Comparison of cross-sections of (A, C, E) Normal anthers and (B, D, F) HT-ms anthers.
Cross-sections stained with Safranin O-Fast Green. Scale bars = 100 μm. T, Tapetum; Tes, tetrads; Msp, microspore; dMsp, Degenerated microspore; MP, mature pollen; E, epidermis; Deh: dehiscence. Cross-section of anthers at: (A, B) tetradstage; (C, D) Binuclear stage; (E, F) Trinucleate stage.

## Slide 2
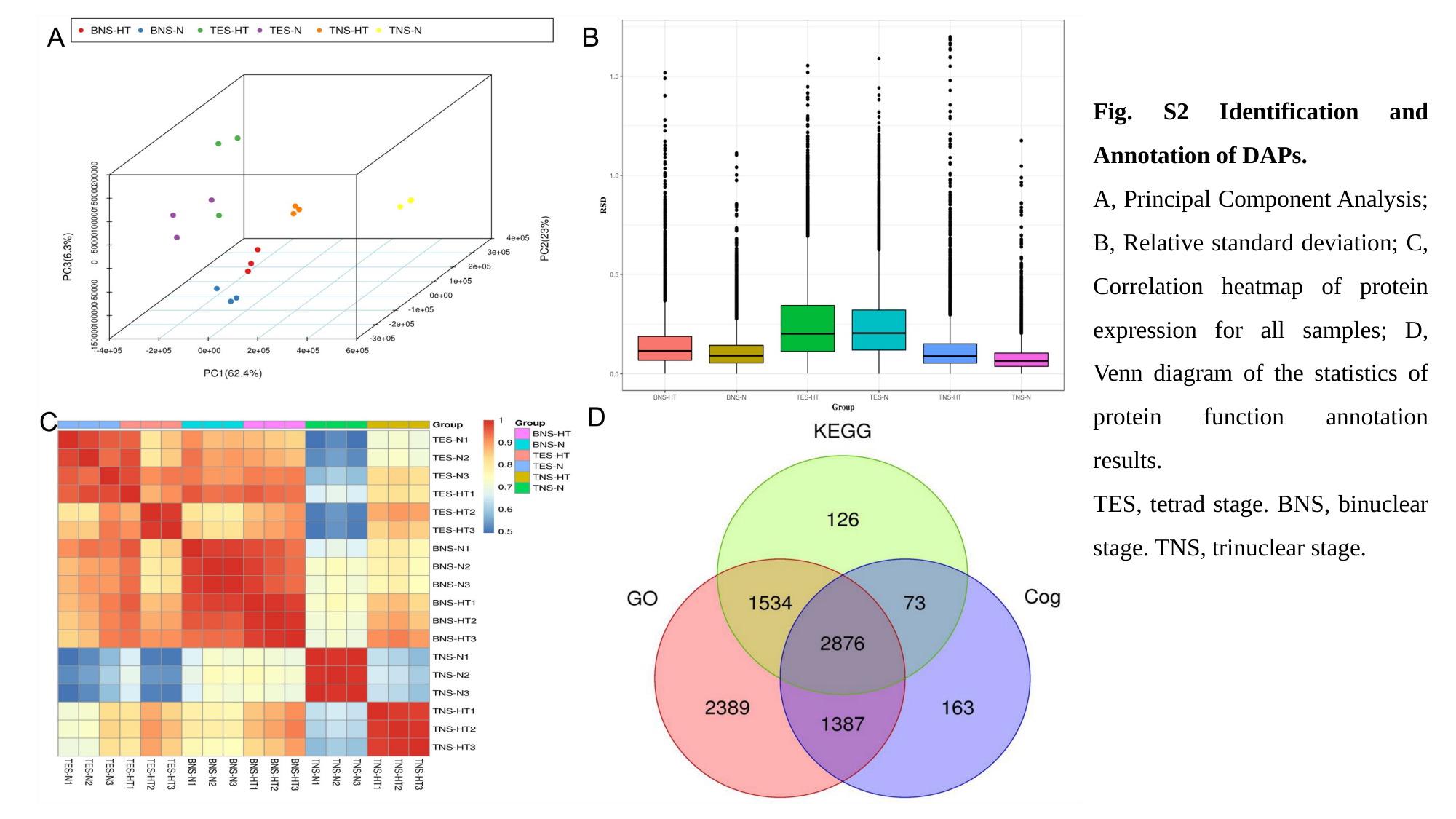

Fig. S2 Identification and Annotation of DAPs.
A, Principal Component Analysis; B, Relative standard deviation; C, Correlation heatmap of protein expression for all samples; D, Venn diagram of the statistics of protein function annotation results.
TES, tetrad stage. BNS, binuclear stage. TNS, trinuclear stage.

## Slide 3
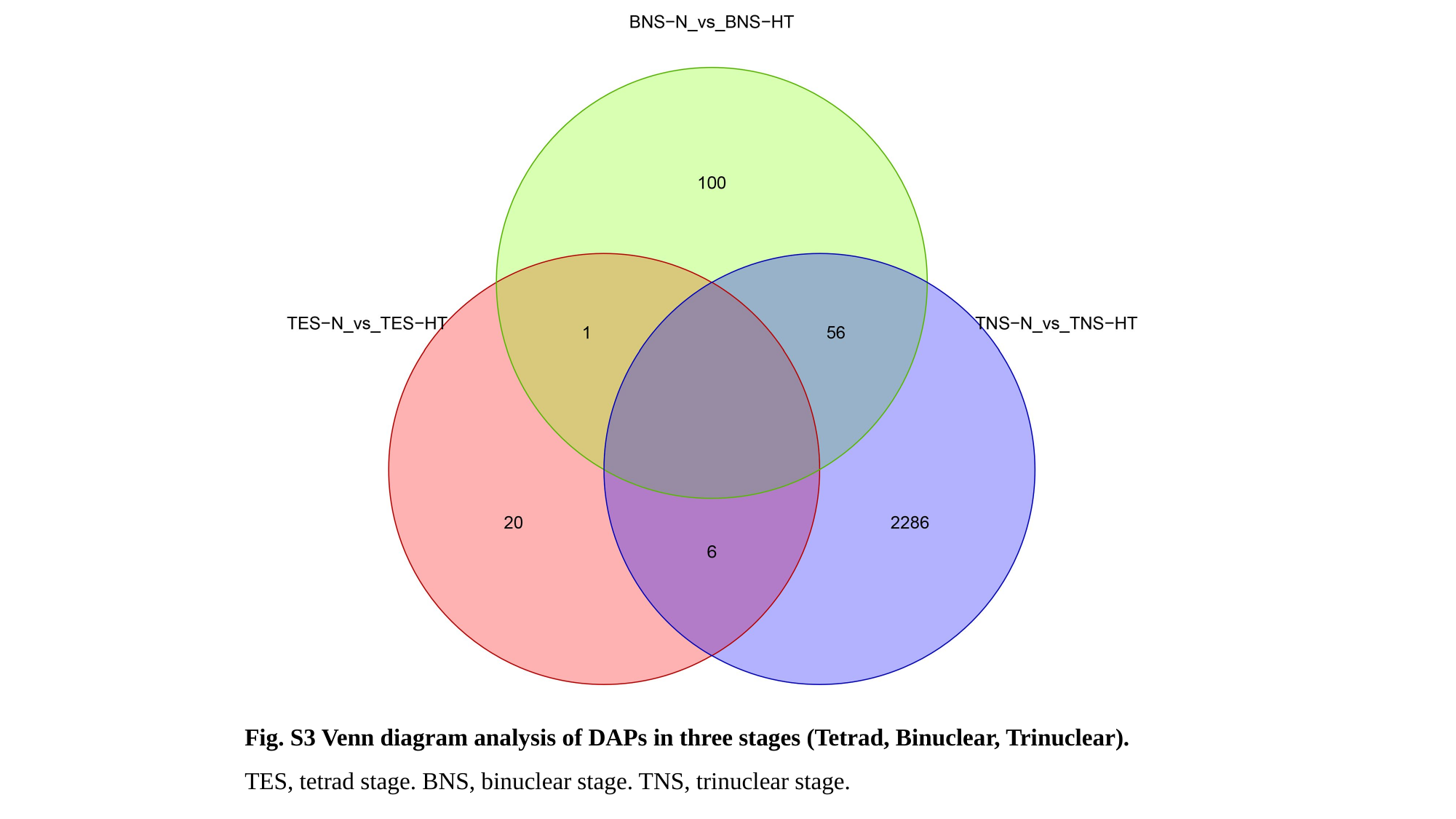

Fig. S3 Venn diagram analysis of DAPs in three stages (Tetrad, Binuclear, Trinuclear).
TES, tetrad stage. BNS, binuclear stage. TNS, trinuclear stage.

## Slide 4
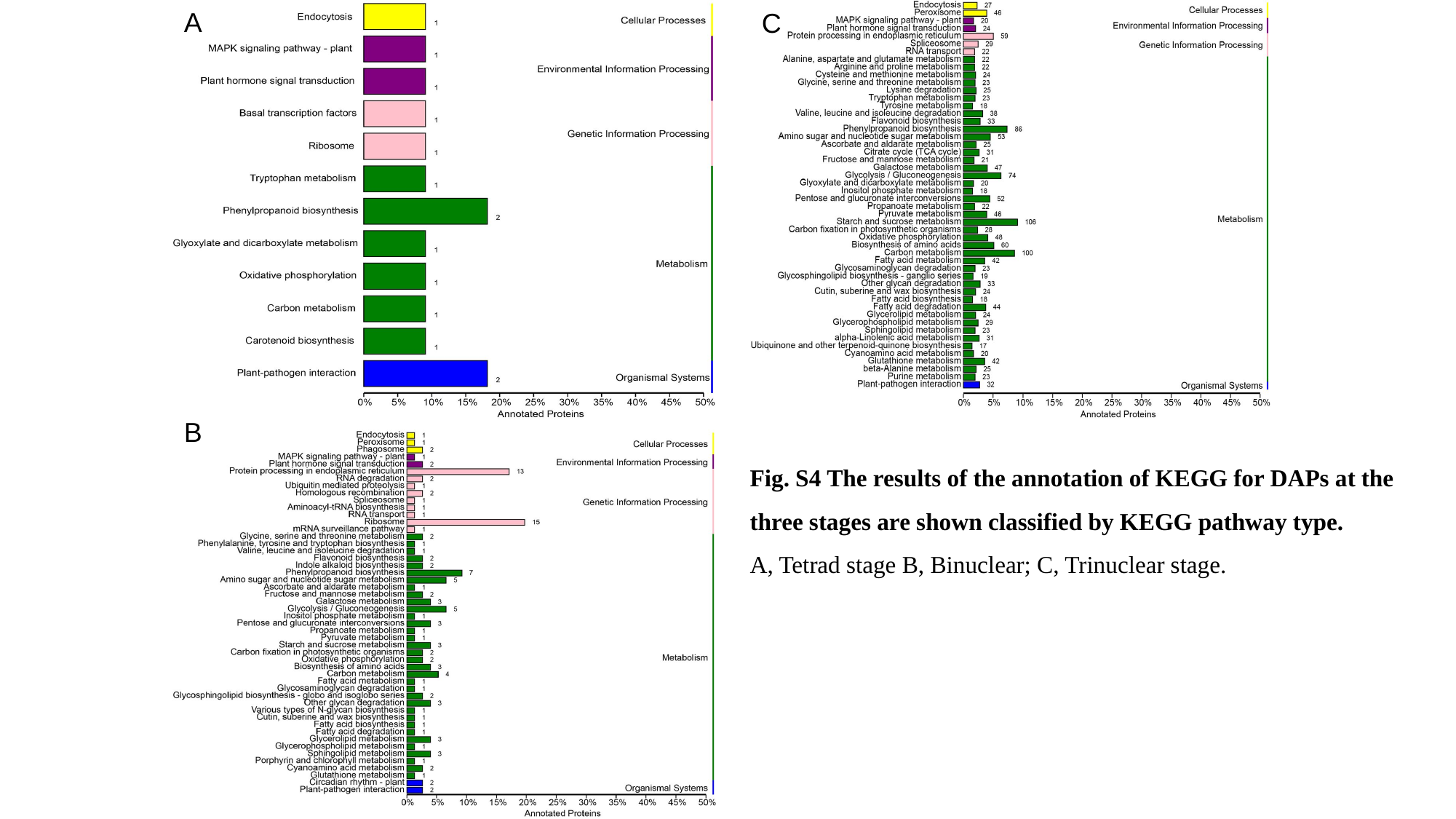

A
C
B
Fig. S4 The results of the annotation of KEGG for DAPs at the three stages are shown classified by KEGG pathway type.
A, Tetrad stage B, Binuclear; C, Trinuclear stage.

## Slide 5
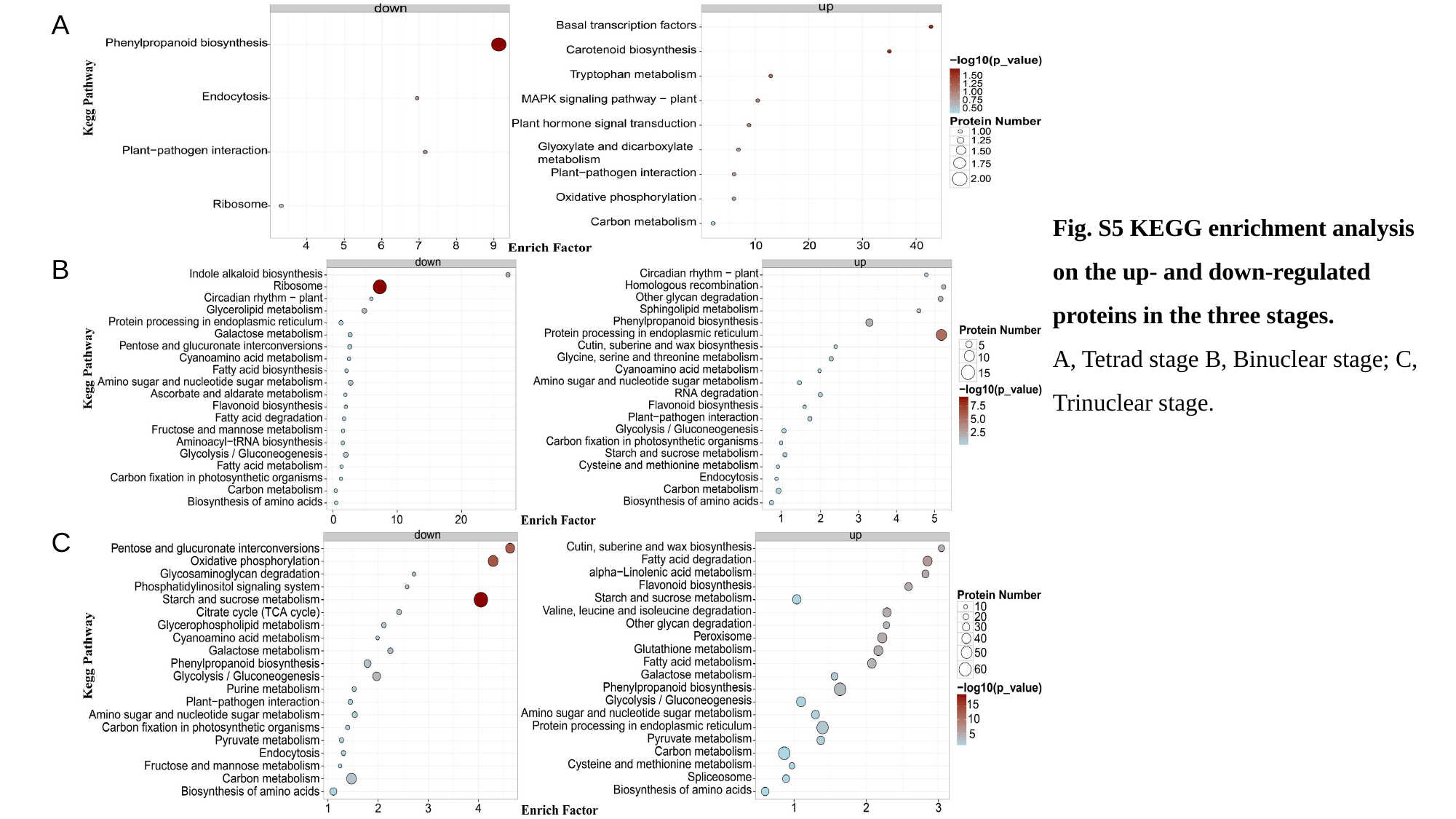

A
Fig. S5 KEGG enrichment analysis on the up- and down-regulated proteins in the three stages.
A, Tetrad stage B, Binuclear stage; C, Trinuclear stage.
B
C

## Slide 6
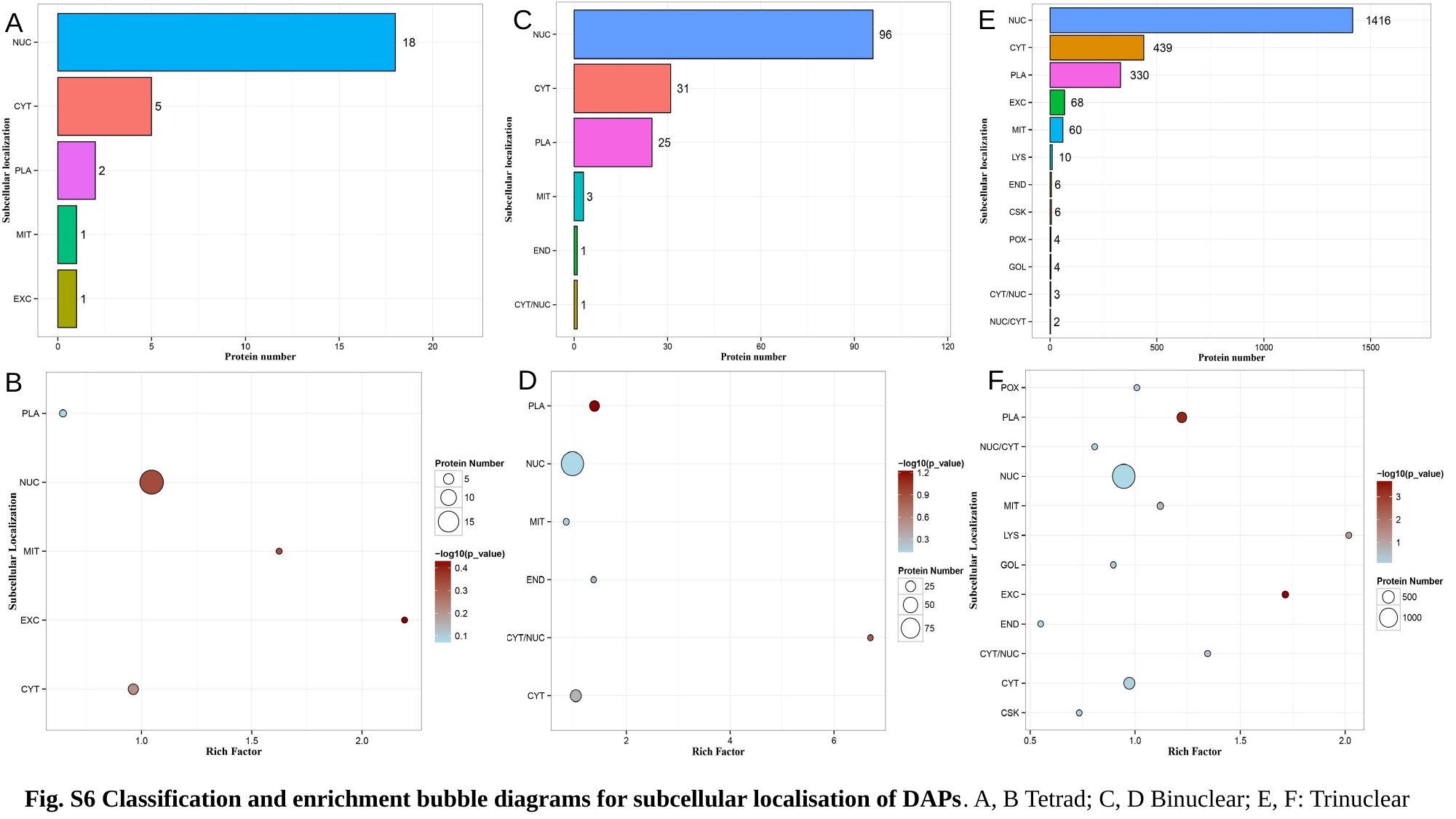

C
E
A
D
F
B
Fig. S6 Classification and enrichment bubble diagrams for subcellular localisation of DAPs. A, B Tetrad; C, D Binuclear; E, F: Trinuclear

## Slide 7
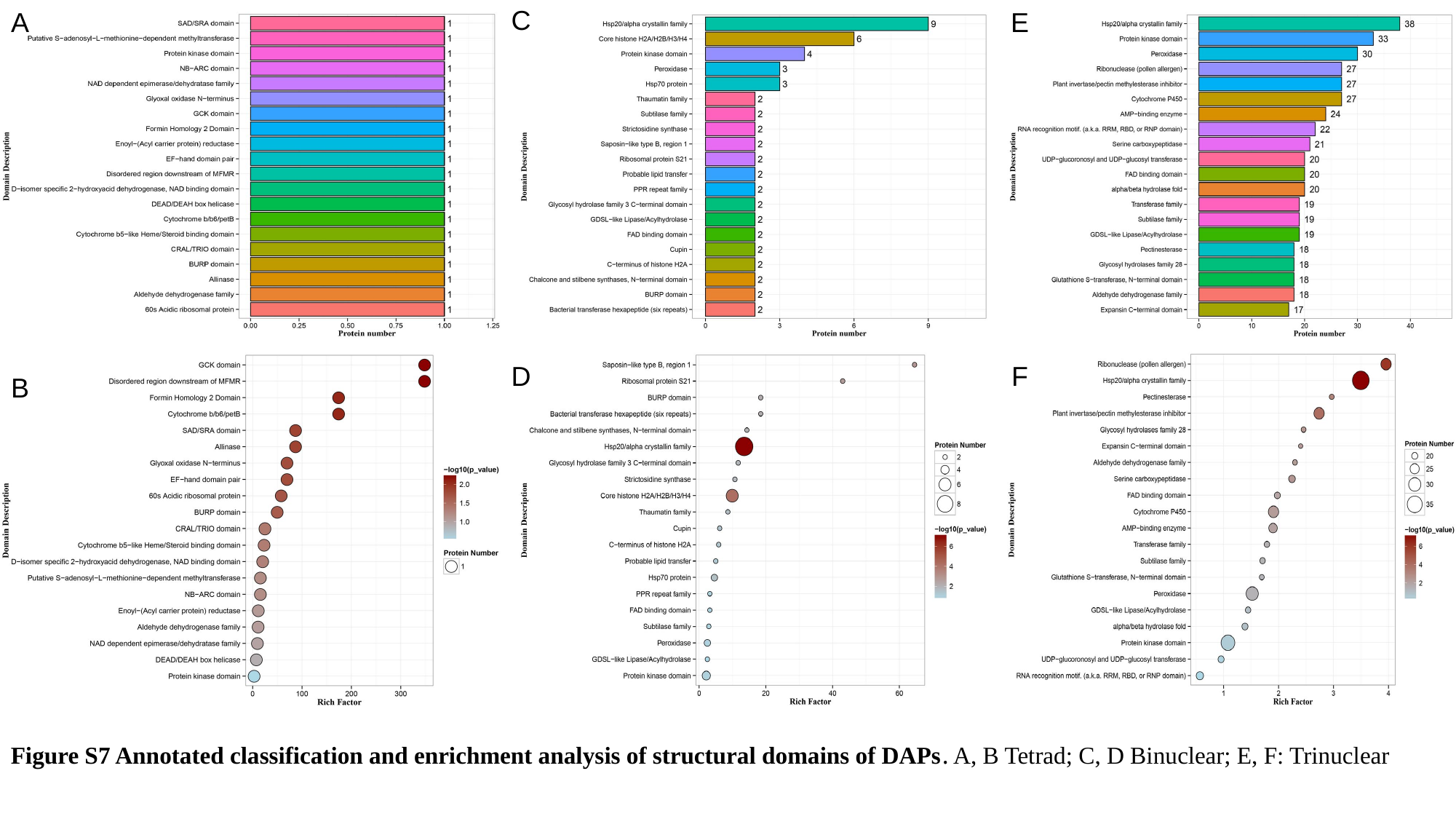

A
E
C
D
F
B
Figure S7 Annotated classification and enrichment analysis of structural domains of DAPs. A, B Tetrad; C, D Binuclear; E, F: Trinuclear

## Slide 8
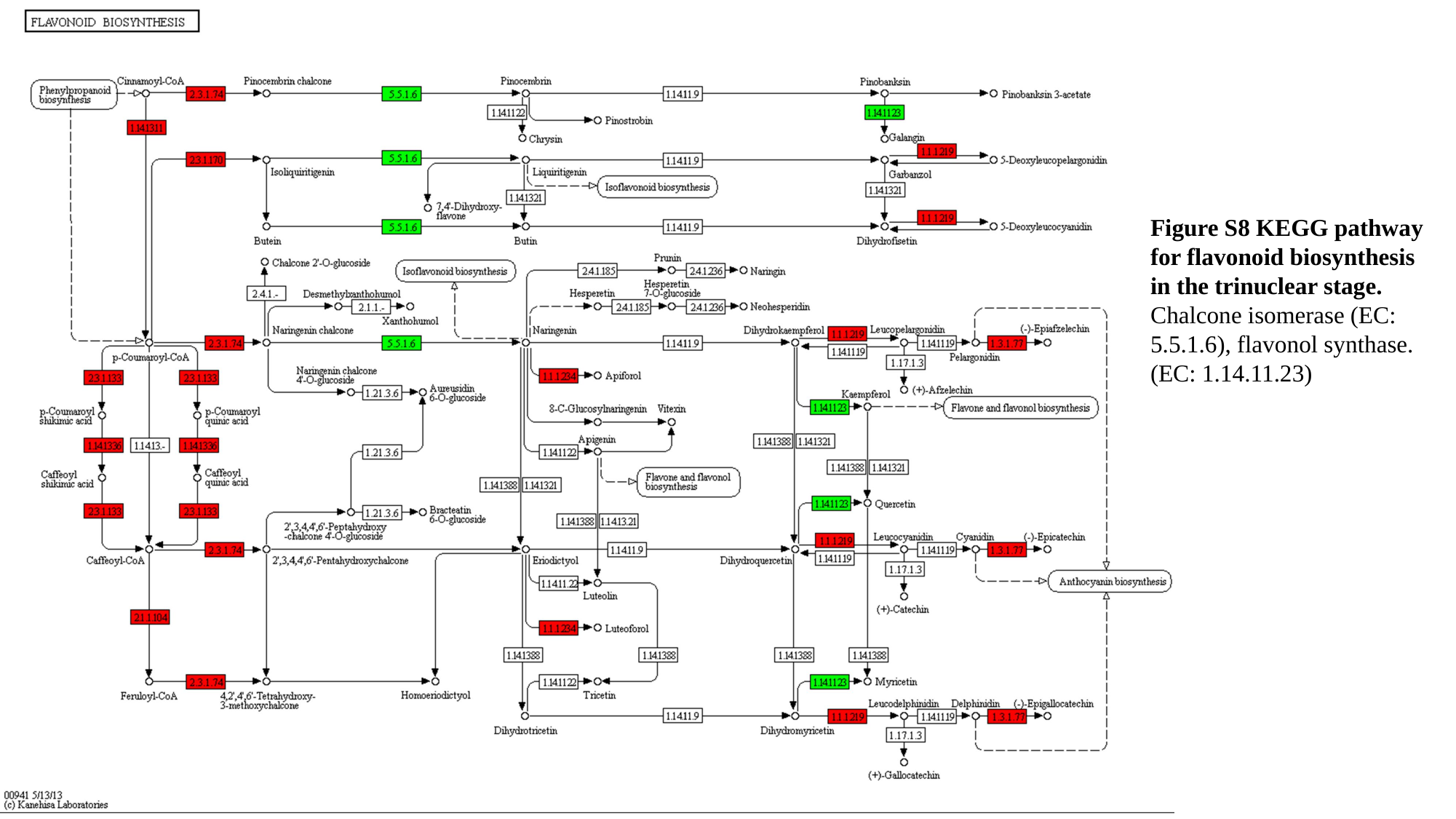

Figure S8 KEGG pathway for flavonoid biosynthesis in the trinuclear stage.
Chalcone isomerase (EC: 5.5.1.6), flavonol synthase. (EC: 1.14.11.23)

## Slide 9
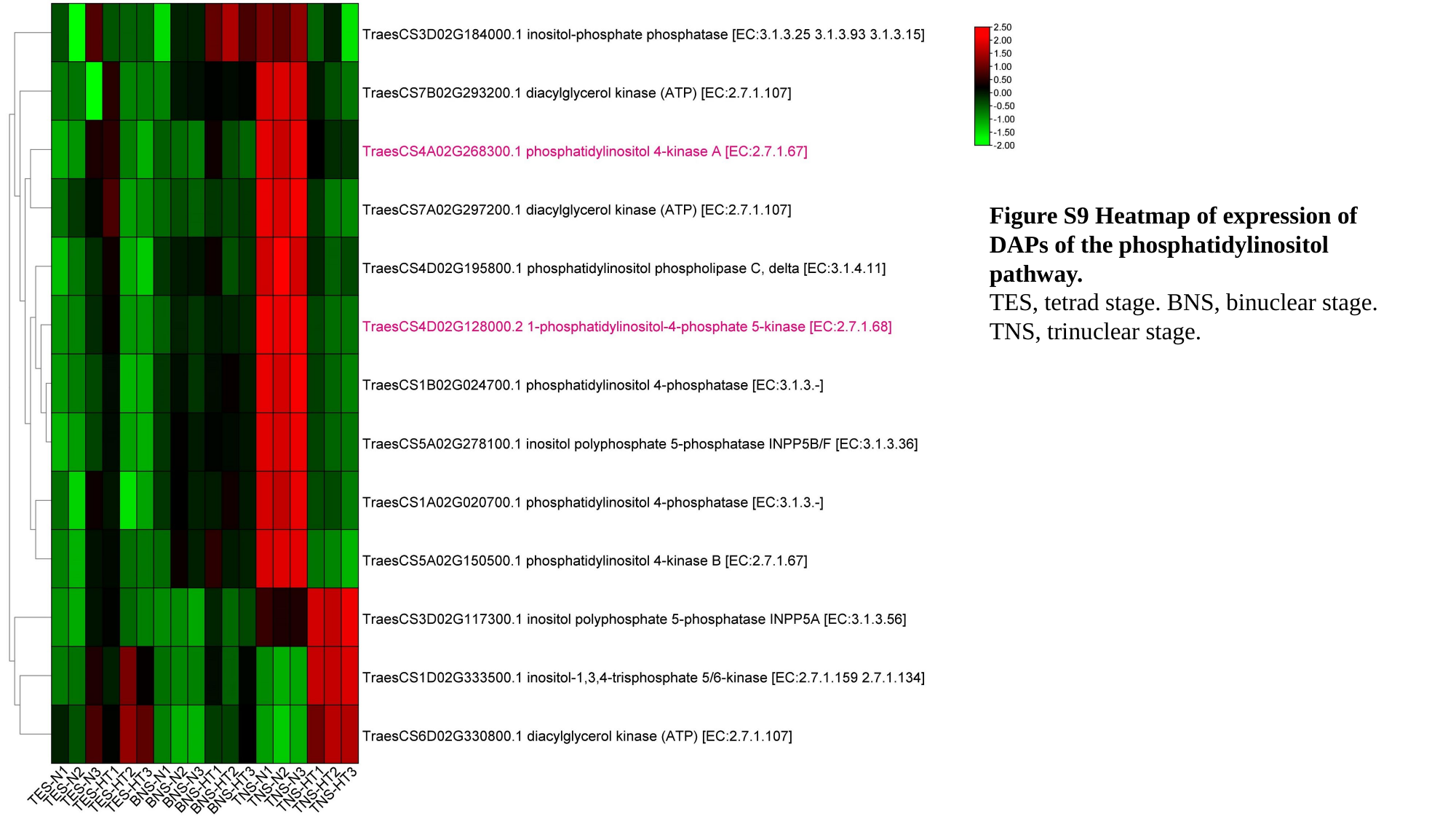

Figure S9 Heatmap of expression of DAPs of the phosphatidylinositol pathway.
TES, tetrad stage. BNS, binuclear stage. TNS, trinuclear stage.

## Slide 10
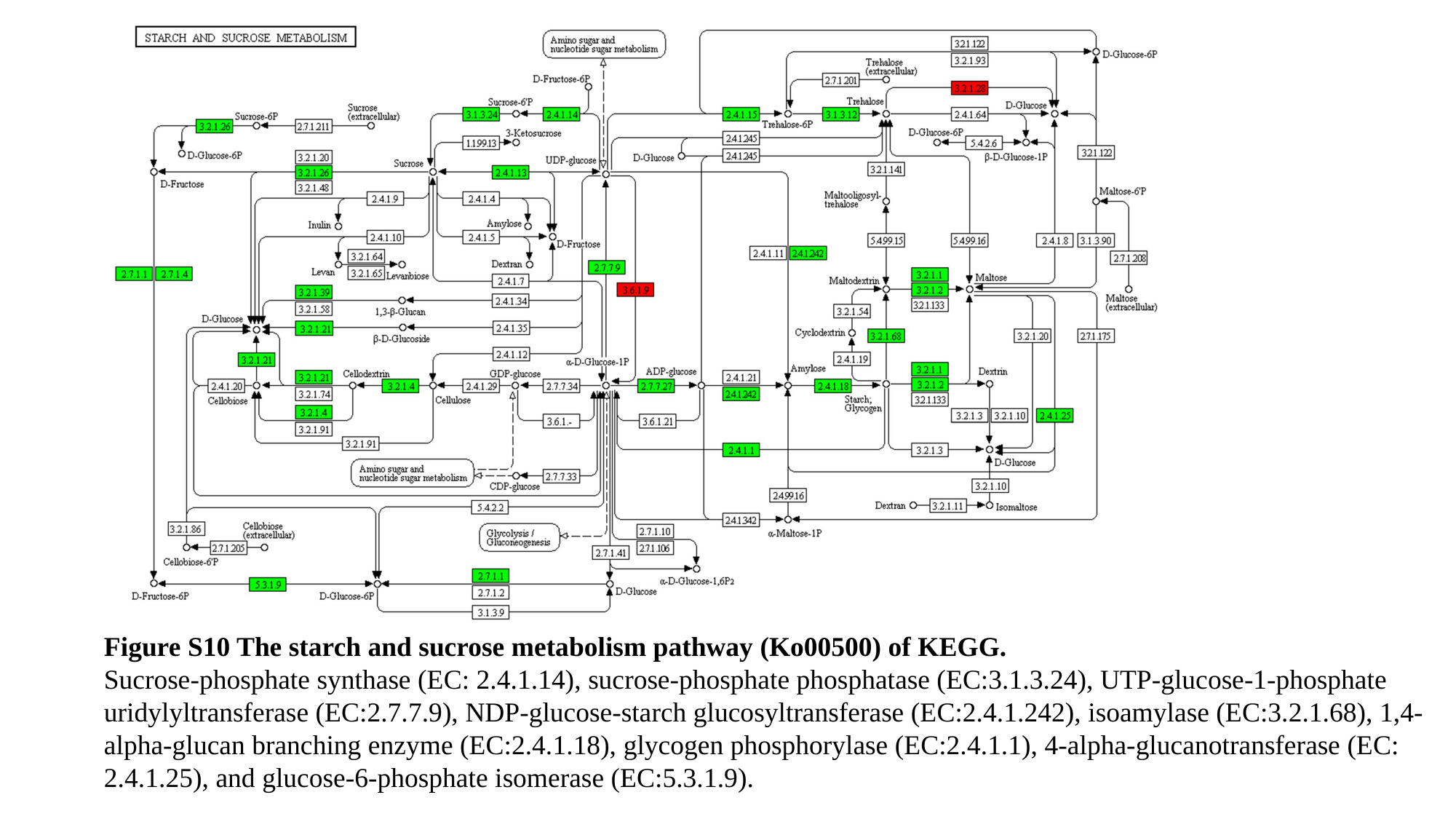

Figure S10 The starch and sucrose metabolism pathway (Ko00500) of KEGG.
Sucrose-phosphate synthase (EC: 2.4.1.14), sucrose-phosphate phosphatase (EC:3.1.3.24), UTP-glucose-1-phosphate uridylyltransferase (EC:2.7.7.9), NDP-glucose-starch glucosyltransferase (EC:2.4.1.242), isoamylase (EC:3.2.1.68), 1,4-alpha-glucan branching enzyme (EC:2.4.1.18), glycogen phosphorylase (EC:2.4.1.1), 4-alpha-glucanotransferase (EC: 2.4.1.25), and glucose-6-phosphate isomerase (EC:5.3.1.9).
